# Nucleic Acids as a Nature‐Inspired Scaffold for Macromolecular Prodrugs of Nucleoside Analogues

**DOI:** 10.1002/advs.201802095

**Published:** 2019-01-28

**Authors:** Franziska Krüger, Vipin Kumar, Pere Monge, Carina Conzelmann, Nikaïa Smith, Kurt V. Gothelf, Martin Tolstrup, Jan Münch, Alexander N. Zelikin

**Affiliations:** ^1^ Institute of Molecular Virology Ulm University Medical Center 89081 Ulm Germany; ^2^ Department of Chemistry and iNano Interdisciplinary Nanoscience Centre Aarhus University Aarhus C 8000 Denmark; ^3^ Aarhus University Hospital Aarhus N 8200 Denmark

**Keywords:** antivirals, herpes simplex virus, nucleic acids, nucleoside analogues, prodrugs

## Abstract

Macromolecular prodrugs (MP) built on the natural phosphodiester and deoxyribose backbone are developed using marketed antiviral nucleoside analogues. These MP are synthesized using automated synthesis, have defined molecular composition, and have a natural mechanism for drug release. These unique attributes, coupled to the efficient cell entry and potent antiviral effects, position the prodrugs scaffolded on nucleic acids favorably for translational studies.

Nucleic acid scaffolds based on (deoxy)ribose and phosphodiester linkage have evolved as the most reliable tool to store, read, and copy information. In modern days, nucleic acids are also highly warranted in biomedicine, biotechnology, and nanotechnology.^1^, ^2^, ^3^, ^4^ The defining characteristics of these scaffolds are their stability and the availability of methods to precisely control their sequence and therefore the molecular structure and function. Herein, we offer a new prospect on nucleic acids and hypothesize that these form a unique scaffold for macromolecular prodrugs (MP). The latter hold immense promise for targeted drug delivery.^5^, ^6^ However, carbon‐chain MP thus far have failed in clinical trials and are disadvantaged due to the nondegradable nature of the polymer backbone and the inevitable dispersity of chains by molar mass meaning inhomogeneity of the formulation. The main‐chain biodegradable peptide or polysaccharide scaffolds present viable alternatives and indeed the current front‐runner MP in translational studies is based on poly(glutamic acid).^6^ Herein, we propose that superior MP can be engineered on the nucleic acid scaffold, specifically for nucleoside analogues (**Figure** **1**). The latter comprise a highly important class of drugs with approved applications in anticancer^7^ and antiviral^8^ therapies. Nucleic acid scaffolds offer a fully natural mechanism for degradation and drug release. They can be engineered to have the highest possible deliverable payload (each monomer unit can contain a therapeutic nucleoside), possibly comprised of different nucleoside analogues to make up a combination therapy. Existing prior art is limited to the nucleic acids containing 5‐fluorouracil and the registered antiproliferative effects thereof.^9^, ^10^ Beyond these reports, the landscape of possibilities associated with the nucleic acid scaffolded MP (termed herein “therapeutic nucleic acids,” TNA) is undefined and remains highly appealing.

**Figure 1:**
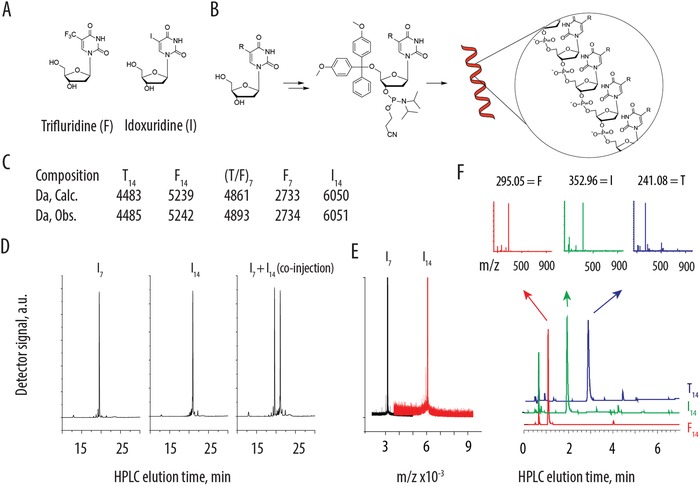
A) Chemical formula of trifluridine and idoxuridine; B) Schematic illustration for the conversion of antiviral drugs into monomers for automated synthesis of nucleic acid (R = I for idoxuridine or R = CF_3_ for trifluridine). C) Mass spectrometry characterization of TNA with regards to the molar mass. D) HPLC characterization of TNA‐I_7_ and TNA‐I_14_. Sequences elute at distinctly different times, as individual peaks, with minimal content of shorter oligomers thus illustrating that TNA are essentially monodisperse reaction products. E) MALDI characterization of the TNA‐I_7_ and TNA‐I_14_ supporting the notion that TNA are obtained as monodisperse reaction products. F) LC–MS analyses illustrating that upon exhaustive digestion (phosphodiesterase I from *Crotalus adamanteus* venom), TNA release the expected idoxuridine, trifluridine, or thymidine.

In this work, we designed TNA using idoxuridine, an approved drug against herpes simplex virus (HSV‐1/2),^8^ and trifluridine, an anticancer agent (Lonsurf) also used in antiviral therapy^8^ (Figure 1A). Synthesis of TNA was developed such as to capitalize on the existing methodology for automated syntheses of nucleic acids. Toward this end, trifluridine and idoxuridine were first converted to the 4,4′‐dimethoxytrityl (DMT)‐protected phosphoramidite derivatives (Figure 1B, for details on synthesis see Supporting Information). Resulting TNAs were either 7‐mers or 14‐mers with sequences containing only the antiviral drug, or the antiviral drug and thymidine in an alternating sequence. The latter were designed for a broader understanding of the structure–activity relationship of TNAs. Products were characterized by mass spectrometry, HPLC, and MALDI to confirm purity and composition (Figure 1C–E). These analyses demonstrate that the synthesized MP were essentially monodisperse as chemical entities with both the composition and the degree of polymerization precisely controlled by the synthesis. This comes in stark contrast to the conventional MP obtained through, radical polymerization techniques^11^, ^12^ in which case dispersity of chains is inevitable and the resulting polymers are an ensemble of products rather than a molecularly defined structure.^13^ TNAs are therefore advantageous from the perspective of translational potential since FDA imposes increasingly tighter regulations on polymers for drug delivery. Finally, to illustrate the natural degradation mechanism for drug release, the TNAs were exhaustively digested using a nuclease/phosphodiesterase enzyme. LC–MS analyses revealed that degradation of TNA affords the expected nucleosides (thymidine, trifluridine, or idoxuridine) as the main product of natural oligomer decomposition (Figure 1F), thus providing the final element of validation of the composition for TNA.

TNA engineered as macromolecular prodrugs, unlike antisense oligonucleotides, siRNA, and other nucleic acid based therapeutic molecules, do not have to stay intact for cytosolic intracellular delivery but in fact must be degraded to elicit their therapeutic effect, **Figure** **2**A. Nuclease‐mediated processing of TNA can occur intracellularly and/or extracellularly (either way contributing to the overall natural drug release from the prodrug). To investigate this, we used HPLC quantification to monitor the intact TNA and the released idoxuridine. We observed that TNA‐I_14_ is stable in serum‐free cell culture medium over at least 24 h but undergoes a natural degradation in the presence of serum, Figure 2B. We observed disappearance of the full‐length oligonucleotide already within 2 h of incubation of TNA in serum (data not shown). However, during this time, we documented only a minor concentration of the released idoxuridine, Figure 2C, which suggests preprocessing of TNA into shorter oligomer species during this time. Idoxuridine release became prominent with incubation times between 2 and 6 h.

**Figure 2:**
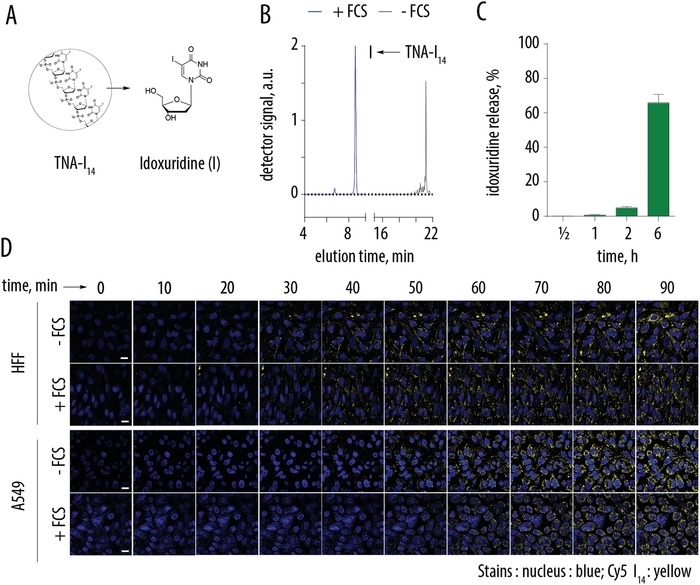
A) Schematic illustration of the nuclease‐mediated degradation of the TNA resulting in the release of idoxuridine. B) HPLC elution profiles for TNA‐I_14_ after a 24 h incubation in cell culture medium with or without FCS. C) HPLC‐based quantification of the idoxuridine release from TNA‐I_14_ upon its incubation in serum‐containing cell culture medium. Presented results are mean of three independent experiments ± S.D. D) Time‐lapse confocal laser scanning microscopy images illustrating cell entry for TNA‐I_14_ in the presence of FCS (DMEM containing 10% FCS) or absence of FCS (X‐VIVO medium) in human foreskin fibroblasts (HFF) and lung carcinoma cell line (A549). Nuclei were stained with Hoechst 33 342; TNA‐I_14_‐Cy5 is false‐colored in yellow. Scale bar (same for all panels): 20 µm.

Independently, we monitored the kinetics of TNA cell entry using time‐lapse confocal laser scanning microscopy. Cell entry was fast and clearly observable already within 20–30 min of TNA incubation with the human foreskin fibroblast cells, and within 50–60 min for A549 lung cancer cells, Figure 2D. Furthermore, rate of cell entry for the TNA was independent of the presence of serum, that is, independent of TNA degradation. Together, data in Figure 2 strongly suggest that TNA may undergo initial scission in the extracellular space in the presence of serum, but this process is not a strict requirement for cell entry, and TNA exhibit fast translocation into cells irrespective of this preprocessing. This conclusion agrees well with prior data on the subject illustrating that oligonucleotides of this length exhibit efficient cell entry via endocytosis, possibly aided by specific membrane‐bound proteins.^14^, ^15^

To determine the antiviral effects of the synthesized TNA, as well as idoxuridine and trifluridine as controls, we used a luminescence‐based cell viability assay that allows quantification of the HSV‐2 induced cytopathic effects and its inhibition by antivirals (**Figure** **3**A). TNA based on thymidine, TNA‐T_14_, elicited a surprising antiviral effect, which was nevertheless characterized by low potency and moderate efficacy of treatment. In turn, the trifluridine‐based TNA‐F_14_ proved to have cell growth inhibitory effects, which is not surprising given that trifluridine is a marketed anticancer agent. Interestingly, the TNA of alternating T and F, (T/F)_7_, did not affect HSV‐2 infection, and also did not show any cytotoxic effects. Most importantly, TNAs based on idoxuridine proved to be potent, efficacious inhibitors of HSV‐2, with virtually no toxicity. Strikingly, activity‐related IC_50_ for TNA based on idoxuridine (75 nm for TNA‐I_14_, 110 nm for TNA‐I_7_, and 263 nm for TNA‐(T/I)_7_ expressed in molarity of the nucleoside) was superior to that of the parent drug (26 µm). In other words, TNA‐I_14_ was ≈35‐fold more potent than idoxuridine when expressed in molarity of the incorporated drug, and more potent than idoxuridine even if expressed in molarity of TNA chains. This phenomenon cannot be explained by the extracellular drug release, in which case the best outcome would be a matched potency of the prodrug and the drug. We believe that enhanced potency of the TNA is therefore due to facilitated cell entry for TNA (Figure 2D),^14^, ^15^ with ensuing exhaustive intracellular processing of the prodrugs for drug release.

**Figure 3:**
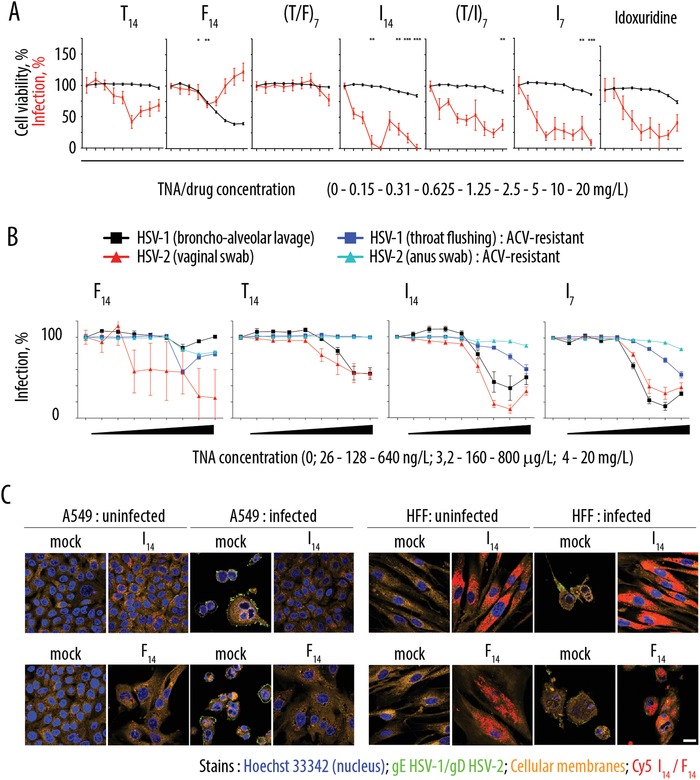
A) Dose response curves for TNA in preventing HSV‐2 infection of Vero E6 cells and corresponding cell viability data. Cells were incubated with TNAs for 2 h prior infection, and 72 h afterward infection was determined by using CellTiter‐Glo (R) (CTG). To determine the cell viability, Vero E6 was incubated with TNAs for 74 h without infection. *n* = 3 in triplicates, ±SEM. *P < 0.1, **P < 0.01, and ***P < 0.001, by one‐way ANOVA with Bonferroni post‐test. B) Dose response curves illustrating activity of TNA against HSV‐1 and HSV‐2 strains sensitive or resistant to acyclovir. Vero E6 cells were incubated with TNAs for 2 h prior infection, infected and 72 h later infection rates were determined by using a cell viability assay. *n* = 3 in triplicates, ±SEM. C) Confocal laser scanning microscopy images illustrating infection of A549 cells with HSV‐1 and HFF cells with HSV‐2 in the presence of TNA‐Cy5‐I_14_ and TNA‐Cy5‐F_14_. After fixation, cells infected with HSV‐1 were stained with an antibody against the glycoprotein E (gE) of HSV‐1 and a secondary antibody labeled with Alexa Fluor 488. The same was done for cells infected with HSV‐2 by using an antibody against the gD of HSV‐2. Nuclei were stained with Hoechst 33 342, cellular membranes with Cell Mask Deep Orange. The nucleic acids are coupled to the fluorophore Cy5. TNA‐Cy5‐I_14_ and TNA‐Cy5‐F_14_ did not show any cytotoxic effects in used cell lines determined with cell viability assay (data not shown). Scale bar (same for all panels): 20 µm.

Encouraged by the above data, we tested TNA in an advanced setting using clinical isolates of HSV‐1 (from bronchoalveolar lavage, BAL) and HSV‐2 (from vaginal swab). TNA based on trifluridine (TNA‐F_14_) revealed virtually no antiviral activity against HSV‐1 and minor, variable antiviral activity against HSV‐2 (Figure 3B). The counterpart based on thymidine (TNA‐T_14_) exhibited modest antiviral effect at highest concentrations. Idoxuridine‐based TNA (TNA‐I_14_ and I_7_) proved to be the lead formulations with potent inhibitory effects against HSV‐1 from BAL and for HSV‐2 from vaginal swab, with IC_50_ values close to those against HSV‐2 shown in Figure 3A. Confocal laser scanning microscopy imaging provided visual illustration of the TNA cell entry and concurrent antiviral effects mediated by these prodrugs (best viewed on human foreskin fibroblast cells, HFF), Figure 3C. While HSV infection led to the loss of the typical fibroblast cell phenotype, exposure to TNA‐I_14_ (but not the inactive and cytotoxic TNA‐F_14_) restored the cell phenotype.

To gain further insights into the antiviral mechanism, we used acyclovir (ACV)‐resistant strains of the viruses (HSV‐1 from throat flushing and HSV‐2 from anus swab), Figure 3B and Figure S3 (Supporting Information). In these strains, ACV resistance is mediated through a frameshift mutation in the viral thymidine kinase,^16^ which renders phosphorylation of ACV and other nucleosides inefficient,^17^, ^18^, ^19^ whereas the virus surface remains unaltered. General polyanion‐mediated inhibition of viral infectivity is achieved by diverse negatively charged polymers through a direct contact with the viral particles^20^, ^21^, ^22^ and for DNA/TNA should be largely sequence‐independent and similar for the HSV‐1/2, resistant to ACV or not. Our experiments revealed that TNA were largely inactive against the ACV‐resistant virus strains, Figure 3B and Figure S3 (Supporting Information). This observation strongly suggests that antiviral effects elicited by the TNA are not due to a polyanion‐mediated inhibition of virus cell entry, but caused by TNA processing and the released drug. Furthermore, TNA‐I_14_ and TNA‐I_7_ were potent and efficacious antiviral agents in serum‐free cell culture conditions that exclude extracellular processing of TNA (Figure S4, Supporting Information), strongly suggesting that TNA processing for drug release is intracellular.

Drug release from TNA may afford nucleosides (that require subsequent kinase activity to have an antiviral effect) or nucleotides (with ensuing kinase‐independent antiviral activity). Since TNA‐I_14_ or I_7_ were not active against the ACV‐resistant strains of HSV (Figure 3B and Figure S3 in the Supporting Information), the activity of the viral kinase likely remained a limiting factor. In other words, our data demonstrate that the intracellular drug release from TNA affords the dephosphorylated nucleoside analogue, idoxuridine.

Taken together, our results indicate that in the presence of serum TNA undergo preprocessing in the extracellular space followed by cell entry and an exhaustive intracellular processing to release the nucleoside antiviral drug. This process results in a remarkable potency of TNA as prodrugs. For in vivo use, stabilization of nucleic acids in circulation can be achieved using diverse methodologies currently in (pre)clinical development for the delivery of small interfering RNA or antisense nucleic acids.^1^ However, our preliminary work suggests that tools of gene transfer such as lipofectamine^1^, ^23^ and the “spherical nucleic acids,” that is, TNA immobilized on gold nanoparticles,^24^, ^25^ do not aid but impeded activity of TNA (see Figures S5–S7, Supporting Information), likely due to restricting the TNA processing. We currently investigate alternative methodologies such as albumin protraction.^26^, ^27^

In this study, we present the first‐in‐class macromolecular prodrugs built on the natural nucleic acid scaffold using marketed nucleoside analogues. TNA have molecularly defined composition, which is a highly favorable attribute concerning the regulatory approval, and release the drug via a natural, nuclease‐mediated mechanism. TNA exhibited fast, unaided cell entry and exerted antiviral effects with high potency superior to that of the parent nucleoside analogues. Together, the natural mechanism of drug release, the perfectly controlled composition, and the high antiviral activity position TNA is highly favorably for translational studies.

## Conflict of Interest

The authors declare no conflict of interest.

## Supporting information

Supplementary

## References

[1] H. Yin , K. J. Kauffman , D. G. Anderson , Nat. Rev. Drug Discovery 2017, 16, 387. 28337020 10.1038/nrd.2016.280

[2] P. D. Howes , R. Chandrawati , M. M. Stevens , Science 2014, 346, 1247390. 25278614 10.1126/science.1247390

[3] F. Pu , J. Ren , X. Qu , Chem. Soc. Rev. 2018, 47, 1285. 29265140 10.1039/c7cs00673j

[4] A. V. Pinheiro , D. Han , W. M. Shih , H. Yan , Nat. Nanotechnol. 2011, 6, 763. 22056726 10.1038/nnano.2011.187 PMC3334823

[5] R. Duncan , Nat. Rev. Drug Discovery 2003, 2, 347. 12750738 10.1038/nrd1088

[6] R. Duncan , M. J. Vicent , Adv. Drug Delivery Rev. 2013, 65, 60. 10.1016/j.addr.2012.08.012 22981753

[7] C. M. Galmarini , J. R. Mackey , C. Dumontet , Lancet Oncol. 2002, 3, 415. 12142171 10.1016/s1470-2045(02)00788-x

[8] E. De Clercq , G. Li , Clin. Microbiol. Rev. 2016, 29, 695. 27281742 10.1128/CMR.00102-15 PMC4978613

[9] W. H. Gmeiner , A. Skradis , R. T. Pon , J. Q. Liu , Nucleosides Nucleotides 1999, 18, 1729. 10474257 10.1080/07328319908044836

[10] W. H. Gmeiner , W. Debinski , C. Milligan , D. Caudell , T. S. Pardee , Future Oncol. 2016, 12, 2009. 27279153 10.2217/fon-2016-0091 PMC4992963

[11] G. Moad , E. Rizzardo , S. H. Thang , Chem. ‐ Asian J. 2013, 8, 1634. 23606667 10.1002/asia.201300262

[12] K. Matyjaszewski , J. Xia , Chem. Rev. 2001, 101, 2921. 11749397 10.1021/cr940534g

[13] M. Barz , R. Luxenhofer , R. Zentel , M. J. Vicent , Polym. Chem. 2011, 2, 1900.

[14] S. L. Loke , C. A. Stein , X. H. Zhang , K. Mori , M. Nakanishi , C. Subasinghe , J. S. Cohen , L. M. Neckers , Proc. Natl. Acad. Sci. USA 1989, 86, 3474. 2726730 10.1073/pnas.86.10.3474 PMC287160

[15] L. A. Yakubov , E. A. Deeva , V. F. Zarytova , E. M. Ivanova , A. S. Ryte , L. V. Yurchenko , V. V. Vlassov , Proc. Natl. Acad. Sci. USA 1989, 86, 6454. 2549537 10.1073/pnas.86.17.6454 PMC297862

[16] F. Morfin , D. Thouvenot , J. Clin. Virol. 2003, 26, 29. 12589832 10.1016/s1386-6532(02)00263-9

[17] J. Piret , G. Boivin , Antimicrob. Agents Chemother. 2011, 55, 459. 21078929 10.1128/AAC.00615-10 PMC3028810

[18] M. Langlois , J. P. Allard , F. Nugier , M. Aymard , J. Biol. Stand. 1986, 14, 201. 3020058 10.1016/0092-1157(86)90004-1

[19] T. H. Bacon , M. J. Levin , J. J. Leary , R. T. Sarisky , D. Sutton , Clin. Microbiol. Rev. 2003, 16, 114. 12525428 10.1128/CMR.16.1.114-128.2003 PMC145299

[20] T. M. Hinton , K. Zuwala , C. Deffrasnes , S. Todd , S. Shi , G. A. Marsh , M. Dearnley , B. M. Wohl , M. Tolstrup , A. N. Zelikin , Adv. Healthcare Mater. 2016, 5, 534. 10.1002/adhm.201500841 26789641

[21] F. Schandock , C. F. Riber , A. Röcker , J. A. Müller , M. Harms , P. Gajda , K. Zuwala , A. H. F. Andersen , K. B. Løvschall , M. Tolstrup , F. Kreppel , J. Münch , A. N. Zelikin , Adv. Healthcare Mater. 2017, 6, 1700748. 10.1002/adhm.201700748 PMC7161897 28945945

[22] A. Vaillant , J. M. Juteau , H. Lu , S. Liu , C. Lackman‐Smith , R. Ptak , S. Jiang , Antimicrob. Agents Chemother. 2006, 50, 1393. 16569857 10.1128/AAC.50.4.1393-1401.2006 PMC1426958

[23] M. B. L. Kryger , S. L. Pedersen , B. M. Wohl , A. N. Zelikin , Chem. Commun. 2016, 52, 889. 10.1039/c5cc08011h 26576493

[24] J. I. Cutler , E. Auyeung , C. A. Mirkin , J. Am. Chem. Soc. 2012, 134, 1376. 22229439 10.1021/ja209351u

[25] N. L. Rosi , D. A. Giljohann , C. S. Thaxton , A. K. R. Lytton‐Jean , M. S. Han , C. A. Mirkin , Science 2006, 312, 1027. 16709779 10.1126/science.1125559

[26] K. Bienk , M. L. Hvam , M. M. Pakula , F. Dagnæs‐Hansen , J. Wengel , B. M. Malle , U. Kragh‐Hansen , J. Cameron , J. T. Bukrinski , K. A. Howard , J. Controlled Release 2016, 232, 143. 10.1016/j.jconrel.2016.04.013 27084489

[27] H. Liu , K. D. Moynihan , Y. Zheng , G. L. Szeto , A. V. Li , B. Huang , D. S. Van Egeren , C. Park , D. J. Irvine , Nature 2014, 507, 519. 24531764 10.1038/nature12978 PMC4069155

