# Supplementary material for: Nucleic Acids as a Nature‐Inspired Scaffold for Macromolecular Prodrugs of Nucleoside Analogues
Source: Adv Sci (Weinh). 2019 Jan 28;6(6):1802095. doi: 10.1002/advs.201802095 (PMC6425433; doi:10.1002/advs.201802095)
Supplement: Supplementary file 1 — Supplementary [file ADVS-6-1802095-s001.pdf]

Copyright WILEY-VCH Verlag GmbH & Co. KGaA, 69469 Weinheim, Germany, 2019.

Supporting Information

for *Adv. Sci.,* DOI: 10.1002/advs.201802095

Nucleic Acids as a Nature-Inspired Scaffold for Macromolecular Prodrugs of Nucleoside Analogues

Franziska Krüger, Vipin Kumar, Pere Monge, Carina Conzelmann, Nikaïa Smith, Kurt V. Gothelf, Martin Tolstrup, Jan Münch, and Alexander N. Zelikin**

Copyright WILEY-VCH Verlag GmbH & Co. KGaA, 69469 Weinheim, Germany, 2016.

Supporting Information

Nucleic Acids as a Nature-Inspired Scaffold for Macromolecular Prodrugs of Nucleoside Analogues

Franziska Krüger,‡ Vipin Kumar,‡ Pere Monge, Carina Conzelmann, Nikaïa Smith, Kurt V. Gothelf, Martin Tolstrup, Jan Münch,* and Alexander N. Zelikin*

F. Krüger, C. Conzelmann, Dr. N.Smith, Prof. J.Münch
Institute of Molecular Virology, Ulm University Medical Center, 89081, Ulm, Germany
E-mail: jan.muench@uni-ulm.de

Dr. V.Kumar, P.Monge, Prof. K. Gothelf, Dr. A.N. Zelikin
Department of Chemistry and iNano Interdisciplinary Nanoscience Centre, Aarhus University, Aarhus C 8000 Denmark
Email : zelikin@chem.au.dk

Dr. M. Tolstrup
Aarhus University Hospital, Aarhus N 8200 Denmark

Synthesis of Phosphoramidite monomers:

For the synthesis of phosphoramidite monomers **5**, and **6**, commercially available Iodoxuridine **1** and Trifluridine **2** nucleosides were used as starting material[1, 2] (scheme 1). Lipoic acid was used for the conjugation of oligonucleotides on gold nanoparticles; therefore, compound **13** was synthesized and installed on 5’ of oligonucleotides.[3]

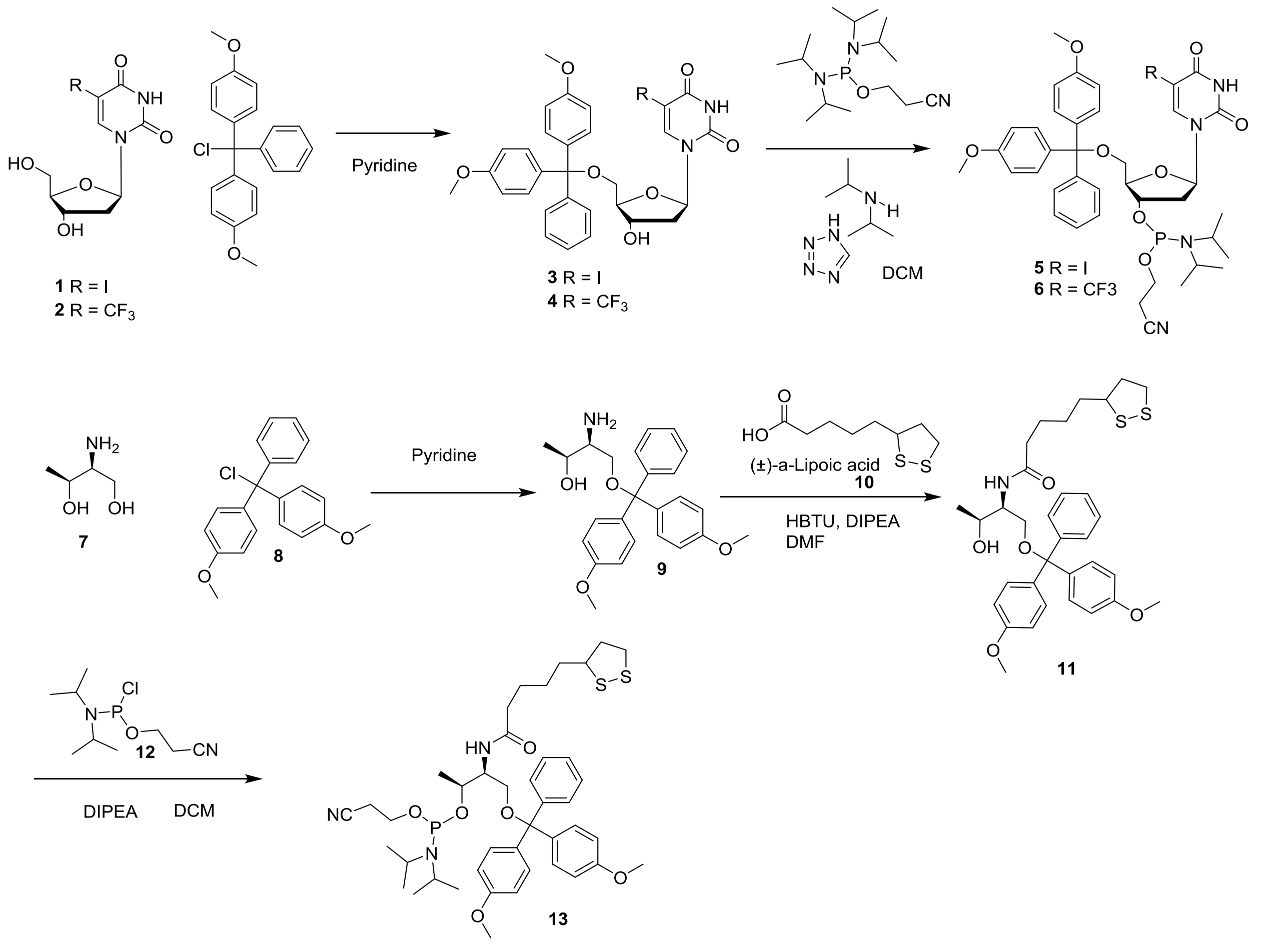

Figure S1. Synthesis of phosphoramidite monomers.

General procedure for DMT protection of 3, 4 and 9 compounds: Starting material (1 equiv.) was dissolved in dry pyridine and DMT-Cl (1.2 equiv.) was added and reaction was stirred overnight under argon condition. Pyridine was evaporated under high vacuum and ethyl acetate was added to oily paste then, washed with sat. $NaHCO_3$ and brine and dried over sodium sulfate. The organic solvent was removed by rotary evaporator and the compound was purified by column chromatography (MeOH/ DCM with 0.5% triethyl amine).

General procedure for phosphoramidite synthesis for 5, 6: Anhydrous dichloromethane was added to the DMT alcohol (**3** or **4,** 1 equiv) followed by the addition of diisopropyl ammonium tetrazolide (0.7 equiv). Subsequently, 2-cyanoethyl N,N,N',N'-tetraisopropylphosphordiamidite (1.3 equiv) was added and allowed to stir at room temperature for 3 hr. The reaction was diluted with dichloromethane and poured in saturated sodium bicarbonate aqueous solution. The product was extracted and dried over dry sodium sulfate. Compound was purified by column chromatography.

1-((2R,4S,5R)-5-((bis(4-methoxyphenyl)(phenyl)methoxy)methyl)-4-hydroxytetrahydrofuran-2-yl)-5-iodopyrimidine-2,4(1H,3H)-dione (3):
Yield: 86%

^{1}H NMR (400 MHz, DMSO) δ 8.02 (s, 1 H), 7.42-7.21 (m, 9 H), 6.90 (d, J = 8 Hz, 4 H), 6.11 (t, J = 8 Hz , 1 H), 4.25 - 4.22 (m, 1 H), 3.90 (q, J = 4 Hz , 1 H), 3.75 (s, 6 H), 3.22 - 3.15 (m, 2 H), 2.27 - 2.16 (m, 2 H). ^{13}C NMR (101 MHz, DMSO) δ 161.0, 158.5, 150.5, 145.2, 144.7, 135.9, 135.8, 130.2, 128.4, 128.1, 127.2, 113.8, 86.3, 85.3, 71, 70.3, 64.2, 55.5, 40.7.

1-((2R,4S,5R)-5-((bis(4-methoxyphenyl)(phenyl)methoxy)methyl)-4-hydroxytetrahydrofuran-2-yl)-5-(trifluoromethyl)pyrimidine-2,4(1H,3H)-dione (4):
Yield: 69%
^{1}H NMR (400 MHz, DMSO) δ H (ppm) 8.07 (s, 1 H), 7.39-7.37 (m, 2 H), 7.31-7.20 (m, 7 H), 6.88 (dd, J = 9.23, 2.64 Hz, 4 H), 4.19 - 4.16 (m, 1 H), 3.95 (q, J = 4.71 Hz, 1 H), 3.73 (s, 6 H), 3.22-3.15 (m, 2 H), 2.34-2.20 (m, 2 H). ^{13}C NMR (101 MHz, DMSO) δ 159.2, 158.1, 149.6, 144.8, 135.4, 129.7, 129.7, 127.8, 127.6, 126.7, 113.2, 86.1, 86.0, 85.8, 70.2, 63.6, 55.0, 55.0, 39.5. ^{19}F NMR (376 MHz, DMSO) δ -61.82.

(2R,3S,5R)-2-((bis(4-methoxyphenyl)(phenyl)methoxy)methyl)-5-(5-iodo-2,4-dioxo-3,4-dihydropyrimidin-1(2H)-yl)tetrahydrofuran-3-yl (2-cyanoethyl) diisopropylphosphoramidite (5):
Yield: 78%
^{31}P NMR (162 MHz, C6D6) δ 148.70, 148.17.
(2R,3S,5R)-2-((bis(4-methoxyphenyl)(phenyl)methoxy)methyl)-5-(2,4-dioxo-5-(trifluoromethyl)-3,4-dihydropyrimidin-1(2H)-yl)tetrahydrofuran-3-yl (2-cyanoethyl) diisopropylphosphoramidite (6):
Yield: 80%
^{31}P NMR (162 MHz, C6D6) δ 149.07, 148.61. ^{19}F NMR (376 MHz, C_6D6) δ -62.78 (dd, J = 8.76, 4.96 Hz, 3F).

(2S,3S)-3-amino-4-(bis(4-methoxyphenyl)(phenyl)methoxy)butan-2-ol (9):
Yield: 89%
^{1}H NMR (400 MHz, DMSO) δ 7.40 (d, J = 7.64, 2 H), 7.32 - 7.19 (m, 7 H), 6.89- 6.86 (m, 4 H), 3.73 (s, 6 H), 3.63 (p, J = 6.07 Hz, 1 H), 3.01 (q, J = 5.38, 1 H), 2.85 - 2.81(m, 1 H), 2.57 (q, J = 5.62 Hz, 1 H), 0.95 (d, J = 6.28, 3 H). ^{13}C NMR (101 MHz, DMSO) δ 158, 145.2, 136, 135.9, 129.7, 127.7, 126.5, 113.1, 85.1, 66.8, 65.4, 56.7, 55, 20.2.

N-((2S,3S)-1-(bis(4-methoxyphenyl)(phenyl)methoxy)-3-hydroxybutan-2-yl)-5-(1,2-dithiolan-3-yl)pentanamide (11): To a stirred solution of **9** (1 equiv) in dry DMF, diisopropylethylamine (3 equiv) was added followed by the addition of **10** (1.2 equiv) and HBTU (1.5 equiv). The reaction was allowed to stir at room temperature. The reaction mixture was diluted with ethyl acetate and washed with saturated bicarbonate solution and water. The organic layer was dried over dry sodium sulphate. The solvent was evaporated and the resulting residues were purified by column chromatography gave 0.742 g (58 % yield) of compound as white foam. ^{1}H NMR (400 MHz, DMSO) δ 7.53 (d, J = 9.11 Hz, 1 H), 7.39 (d, J = 8.01 Hz, 2 H), 7.32 - 7.19 (m, 7 H), 6.87 (d, J = 8.45 Hz, 4 H), 4.55 (d, J = 5.44 Hz, 1 H), 3.97 - 3.87 (m, 2 H), 3.74 (s, 6 H), 3.56 - 3.44 (m, 1 H), 3.18 - 3.04 (m, 3 H), 2.89 - 2.84 (s, 1 H), 2.37 - 2.30 (m, 1 H), 2.16 (t, J = 7 Hz, 2 H), 1.85 - 1.78 (m, 1 H), 1.68 -1.61 (m, 1 H), 1.58 - 1.48 (m, 3 H), 1.36 (q, J = 8.24 Hz, 2 H), 0.96 (d, J = 5.87 Hz, 3 H). ^{13}C NMR (101

MHz, DMSO) δ 172.2, 158.0, 145.2, 135.9, 135.9, 135.9, 129.7, 127.8, 126.6, 113.1, 85.1, 65.1, 65.0, 63.1, 63.0, 56.2, 56.1, 55.0, 53.7, 35.2, 35.2, 34.2, 34.2, 28.4, 28.3, 25.4, 25.3, 20.3, 20.3.

(2S,3S)-3-(5-(1,2-dithiolan-3-yl)pentanamido)-4-(bis(4-methoxyphenyl)(phenyl)methoxy)butan-2-yl (2-cyanoethyl) diisopropylphosphoramidite (13): Anhydrous dichloromethane was added to the **11** (1 equiv) followed by the addition of diisopropyl ethylamine (3.3 equiv). Subsequently, 2-Cyanoethyl N,N-diisopropylchlorophosphoramidite (1.3 equiv) was added slowly and reaction was allowed to stir at room temperature for 2 hr. The reaction was diluted with dichloromethane and poured in saturated aqueous sodium bicarbonate. The product was extracted and dried over dry sodium sulfate. Compound was purified by column chromatography gave 0.546 g (56 % yield) as white solid foam. ^{31}P NMR (162 MHz, CD_3CN) δ 147.67, 147.17.

References:

1. Sheardy, R. D.; Seeman, N. C., J. Org. Chem. 1986, 51, 4301-4303.
2. Gmeiner, W. H.; Pon, R. T.; Lown, J. W., J. Org. Chem. 1991, 56, 3602-3608.
3. Pérez-Rentero, S.; Grijalvo, S.; Peñuelas, G.; Fàbrega, C.; Eritja, R., Molecules 2014, 19, 10495-10523.

Synthesis of oligonucleotides:

Standard protocol of phosphoramidite chemistry was used for synthesis of oligonucleotides in which modified and standard monomers were dissolved (100 mM solution) in anhydrous dichloromethane and anhydrous acetonitrile respectively. Each monomer coupling was performed for 2 min extended time in case of modified monomers. Deprotection and oligonucleotides cleavage from solid support were carried out by 50 mM potassium carbonate in dry methanol for 4 hours at room temperature. Oligonucleotides were purified by Glen-Pak™ DNA cartridge. Purity was analyzed by reverse phase HPLC. Cy5 dye was conjugated with maleimide chemistry and conjugates were purified by reverse phase HPLC. Oligonucleotides were analyzed by liquid chromatography–mass spectrometry (the mass of ON-11 was determined by MALDI-TOF).

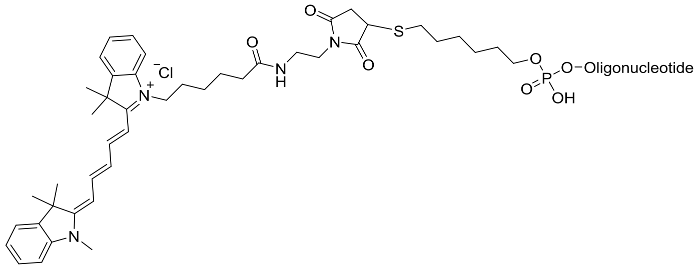

Figure S2. Structure of oligonucleotide with Cy5 conjugated dye at 5'-OH. Fluorescently labelled oligonucleotides contain 14 modifications with Trifluridine or Idoxuridine nucleotides.

Table 1. Results of the LC-MS analyses of the synthesized oligonucleotides.

Code	Sequences		Cal. Mass	Obs. Mass
AZ-VPN-11	5'- TFT FTF TFT FTF TFC-3'	$(T/F)_7$	4861	4893
AZ-VPN-12	5'- FFF FFF FFF FFF FFC-3'	F_{14}	5239	5242
AZ-VPN-13	5'- III III III III IIC-3'	I_{14}	6050	6051
AZ-VPN-14	5'-TIT ITI TIT ITI TIC-3'	$(T/I)_7$	5266	5269
AZ-VPN-15	5'-TTT TTT TTT TTT TTC-3'	T_{14}	4483	4485
AZ-VPN-21	5'- II III IIC-3'	I_7	1568 $(M-2H)^{2-}$	1569 $(M-2H)^{2-}$
AZ-VPN-24	5'- FF FFF FFC-3'	F_7	2733	2734
AZ-VPN-26	5'-TT TTT TTC-3'	T_7	2355	2356
AZ-VPN-	5'- Cy5FFF FFF FFF FFF	Cy_7-F_{14}	6040	6043

27	FFC-3'			
AZ-VPN-29	5'- Cy5III III III III IIC-3'	Cy_7-I_{14}	6851	6854
AZ-VPN-30	5'- lipFFF FFF FFF FFF FFC-3'		5594	5596
AZ-VPN-31	5'- lipIII III III III IIC-3'		6405	6407
AZ-VPN-32	5'-lipTTT TTT TTT TTT TTC-3'		4838	4840

ENZYMATIC DIGESTION OF TNAs

To solutions of the different TNAs (25 μM for I_{14} and 35 μM for F_{14} and T_{14}) in TrisHCl buffer (0.1 M Tris, 0.1 M NaCl, 15 mM $MgCl_2$, pH 8.9) were added Phosphodiesterase I from Crotalus adamanteus venom (Sigma P3134) to a concentration of 35 $mg{\cdot}L^{-1}$ from a stock solution (0.8 $mg{\cdot}mL^{-1}$). The samples were incubated at 37°C for 2h after which samples were spin-filtered through 100 kDa cut-off filters and analyzed by HPLC or by LC-MS. Thymidine 5'P monophosphate (0.4 mM) sample was included in the same conditions to control for background phosphatase activity of the enzyme.

CELL MEDIA NUCELASE ACTIVITY STUDIES ON TNAs

TNAs (I_7, I_{14}, T_{14}) were dissolved in either MEM media or MEM media supplemented with 10% FCS (25 μM). Samples were incubated at 37° C and at given time points aliquots were taken out, spin filtered (10 kDa cut-off, 5000 rcf, 5 min) and analyzed by HPLC a Agilent HPLC equipped with a Phenomenex, Clarity 3μ oligo-RP, 50 x 4.60 mm 3 micro column. Samples I7 and I 14 were monitored at 290 nm, sample T14 at 260 nm. For sample I_{14}, additional quantification of idoxuridine released in the presence of 10% FCS was performed using HPLC in a Shimadzu LC-2010A HT equipped with a Ascentis® Express Peptide ES-C18 column. Quantification was performed in triplicates using a calibration curve of the drug in the same system chromatographic conditions.

Virology experiments

Production of virus and cultivation

HSV-1 and 2 isolates were kindly provided by Prof. Michel (Institute of Virology, Ulm University Medical Center). Acyclovir-resistant strains carry frameshift mutations in the thymidine kinase. HSV-2 eGFP (333) was kindly provided by P. G. Spear, Department of Microbiology-Immunology, Northwestern University, Chicago, USA. All viruses were produced by infecting Vero E6 cells and harvesting of supernatants 48 hours later. To remove cells debris, supernatants were centrifuged at 300 g for 5 min. Virus stocks were stored at -80°C. Cultivation of Vero E6 cells was performed high-glucose Dulbecco's Modified Eagle Medium (DMEM) with 2 mM L-glutamine, 100 units/ml penicillin, 100 µg/ml streptomycin, 1 mM sodium pyruvate and 1x non-essential amino acids (Invitrogen, Glostrup, Denmark) supplemented with 2.5% (v/v) heat-inactivated fetal calf serum (FCS). Human lung epithelial carcinoma cells (A549; ATCC® CCL-185™) and human foreskin fibroblast (HFF; kindly provided by Jens von Einem) were cultured in high-glucose Dulbecco's Modified Eagle Medium (DMEM) with 2 mM L-glutamine, 100 units/ml penicillin, 100 µg/ml streptomycin supplemented with 10% (v/v) heat-inactivated fetal calf serum (FCS).

Dissecting the uptake of therapeutic nucleic acids

To investigate the uptake of therapeutic nucleic acids, 150,000 lung epithelial carcinoma cells (A549) and human foreskin fibroblast (HFF) cells were seeded in 8-well Ibidi slide in 150 µl. The next day, supernatant was discarded and 135 µl fresh medium was added. As medium either X-VIVO™ 15 Serum-free Hematopoietic Cell Medium (Lonza) or high-glucose Dulbecco's Modified Eagle Medium (DMEM) both supplemented with 2 mM L-glutamine, 100 units/ml penicillin and 100 µg/ml streptomycin was used. Additionally, DMEM was supplemented with 10% (v/v) heat-inactivated fetal calf serum (FCS). To stain the nuclei and the plasma membrane the media furthermore contained Hoechst 33342 (Thermo Scientific™) 1:2000 (v/v) and Cellmask Deep Orange plasma membrane stain (Invitrogen) diluted 1:1,000 (v/v). Right before starting the measurement 15 µl of the Cy5-labelled TNA-I_{14} was added resulting in an endconcentration of 20 mg/l on the cells. Image acquisition was performed with Zeiss LSM 719 every 10 min for 10 cycles. Images were processed with Fiji (Nature Methods **2012**, 9(7), 676-682) on the Quadro P6000.

Determination of infection by cytopathic effect and cytotoxicity of therapeutic nucleic acids using a CellTiter-Glo® (CTG) luminescent cell viability assay

To determine the antiviral and cytotoxic effects of therapeutic nucleic acids, 6,000 Vero E6 cells per well were seeded in 96-well flat bottom plate in 100 µl medium. The next day, medium was removed and 70 µl fresh medium was added. TNA and controls were diluted in PBS twofold, starting with a concentration of 200 mg/L. 10 µl were added to cells and incubated for 2 h in CO_2 incubator at 37°C. Cells were then infected with an MOI of 0.02 of HSV-2 eGFP (333) or 20 µl fresh medium was added. 72 h later the ATP levels were quantitated. Therefore, the plates were exposed to room temperature. After 15 min 50 µl of

buffer-substrate-mix of CellTiter-Glo® (CTG) luminescent cell viability assay was added to each well. After 10 min incubation in the dark, the solution was transferred into white microtiter plate and ATP-dependent luminescence was measured using Orion II microplate luminometer (Software Simplicity, 0.1 s measurement). Infection rates were determined by subtracting sample values from untreated control and untreated control was set to 100%.

Determination of infection by cytopathic effect and cytotoxicity of compounds using a Colorimetric 3-(4, 5-dimethylthiazol-2-yl)-2,5-diphenyltetrazolium bromide (MTT)-based cell viability assay

The inhibitory effect of therapeutic nucleic acids against clinical isolates of HSV-1 and HSV-2 was determined by incubating 6,000 Vero E6 cells seeded the day before in 96-well flat bottom plate in 100 µl medium. The supernatant was removed, 70 µl fresh medium and 10 µl of five-fold diluted therapeutic nucleic acids and respective controls starting with a concentration of 200 mg/l was added. 2 h later cells were infected with 20 µl of clinical isolates with an MOI of 0.02. 72 h afterwards, bright field images were taken with a Leica MC120 HD colour camera connected to a microscope (Europa Promotion Leica DM IL LED, Leica microsystems) with a 10x objective. Subsequently, supernatant was removed and 100 µl of 1 to 10 diluted MTT (5 mg/ml) solution was added. After 2.5 h at 37°C supernatant was discarded and formazan crystals were dissolved in 100 µl 1:1 DMSO-EtOH solution. Absorption was measured at 450 nm and baseline was corrected at 650 nm using a Vmax kinetic microplate reader. To determine infection rates, sample values were subtracted from untreated control and untreated control set to 100%.

The same was done for therapeutic nucleic acids conjugated to gold nanoparticles. Gold nanoparticles were dissolved five-fold starting with a concentration of 20 mg/l. Infection was carried out with 20 µl of HSV-2 eGFP (333) and an MOI of 0.02. The determination of infection by cytopathic effect was done as indicated above.

To assess the extracellular stability of the therapeutic nucleic acids 12,000 Vero E6 cells were seeded in 96-well flat bottom plate in 100 µl medium. The next day, medium was removed, cells were carefully washed with PBS and 70 µl X-VIVO™ 15 Serum-free Hematopoietic Cell Medium (Lonza) supplemented with 2 mM L-glutamine, 100 units/ml penicillin, 100 µg/ml streptomycin, 1 mM sodium pyruvate and 1x non-essential amino acids (Invitrogen, Glostrup, Denmark) was added. 72 h post infection in X-VIVO™ medium, following procedure is equal to the one indicated before using therapeutic nucleic acids, an infection with clinical isolates and determination of infection by cytopathic effect using MTT.

Determination of uptake of therapeutic nucleic acids and inhibitory effect against clinical isolates in human cell lines

To investigate the uptake of therapeutic nucleic acids, 40,000 lung epithelial carcinoma cells (A549) and human foreskin fibroblast (HFF) cells were seeded in 8-well Ibidi slide in 150 µl. The next day, supernatant was discarded and 120 µl fresh medium was added. Cy5-labelled therapeutic nucleic acids were diluted 5 fold, starting with a concentration of 200 mg/l, incubated for 2 h at 37°C and subsequently, cells were infected with 15 µl with HSV-1 isolate from broncho-alveolar lavage (A549) or HSV-2 isolate from vaginal swab (HFF) with an MOI 0.02. After 72 h hours at 37°C, supernatant was discarded and cells were fixed with 4%

paraformaldehyde for 10 min at 4°C. All staining steps were carried out at room temperature and between every step, cells were washed twice with PBS. Firstly, a blocking solution containing PBS with 10% (v/v) bovine serum albumin (BSA) and 5% (v/v) FCS was added for 30 min. To visualize the infection with HSV, cells infected with HSV-1 were incubated with a primary antibody against gE (sc-58153; Santa Cruz Biotechnology, Inc.) diluted 1 to 500 (v/v) and cells infected with HSV-2 were incubated with a primary antibody against gD (ab6507; abcam®) diluted 1 to 3,200 (v/v) for 45 min. As secondary antibody Alexa Fluor 488 goat anti-mouse IgG (Invitrogen) was used 1 to 1,000 (v/v) diluted and incubated for 45 min at. To stain the cell nucleus Hoechst 33342 (Thermo Scientific™) 1:2000 (v/v) for 30 min was used and afterwards plasma membrane was stained with Cellmask Deep Orange plasma membrane stain (Invitrogen) diluted 1 to 1,000 (v/v) for 5 min. Cells were stored in PBS, kept in the dark and left at 4°C until microscopy image acquisition using a Zeiss LSM 719 and ZEN software 2.3 blue lite (2011) for image processing.

Transfection of Vero E6 cells with Lipofectamine

6,000 Vero E6 cells were plated into 96 well plate. The next day, therapeutic nucleic acids were prepared with a maximum amount of 1 or 10 µg and TNAs were combined with Lipofectamine 2000 according to the protocol from Invitrogen by life technologies (Protocol Pub. No. MAN0007824 Rev.1.0). Briefly, per each well 0.5 µL of Lipofectamine was diluted into 5µL of Opti-MEM (ThermoFisher) serum free media. The TNAs were also diluted into 5µL of Opti-MEM to have 1 or 10 µg of TNAs final per well. The two solutions were then mixed together and kept at RT for 5 min. Cells were incubated with the mixture for 2 h at 37°C before being washed and infected with HSV-2 (333) eGFP. For a read-out after 24 h an MOI of 0.02 and for a read-out after 72 h an MOI of 0.0003 was used. Infection efficiency was determined by analyzing GFP positive cells by flow cytometry (FACSCanto II, BD).

Statistical analyses

One-way ANOVA followed by Bonferroni's multiple comparison test (*p<0.1,**p< 0.01, ***p< 0.001, ****p< 0.0001) was performed using GraphPad Prism version 7.05 for Windows, GraphPad Software, La Jolla, CA, USA, www.graphpad.com.

Preparation of TNA-coated gold nanoparticles

Stocks solutions of lipoic acid-terminated TNAs in milliQ water were diluted in a suspension of gold nanoparticles (10 nM), to give a final concentration of 3 □M DNA. After incubating overnight, the solutions were brought to a concentration of 0.01% SDS. Then, small amounts of a 4M NaCl solution were added at regular periods of time to bring the final concentration of NaCl to 0.3 M over the course of 8 hours. Particles were incubated in this conditions for 24h, after which they were washed with 4 consecutive centrifugation steps (20000 rcf, 25 min) with milliQ water and resuspended in milliQ water. Concentration of particles was estimated by the absorption coefficient at 532 nm. The amount of DNA conjugated to the gold nanoparticles was estimated by the UV absorption at 260 nm (Trifluridine and Thymidine nucleotides) or 295 nm (Idoxuridine nucleotides) in the supernatant solutions of the first centrifugation step, after correcting for the absorption of remaining particles in suspension at the respective wavelengths.

Additional data figures

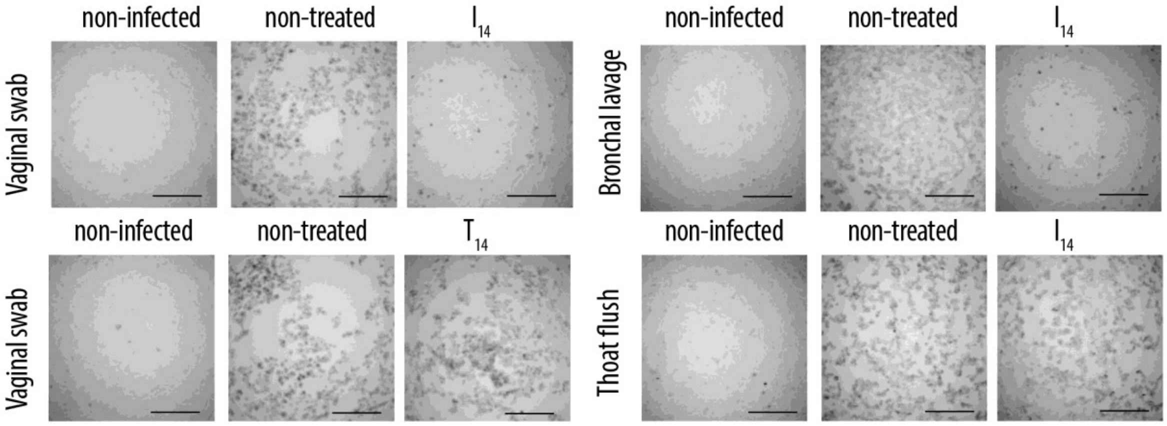

Figure S3. Bright field microscopy images illustrating infection of Vero E6 cells with HSV (from HSV-1 broncho-alveolar lavage (BAL), HSV-2 vaginal swab, acyclovir-resistant HSV-1 from throat flush, acyclovir-resistant HSV-2 from anus swab) in the presence of TNA-I_{14}. Bright field images were taken with a Leica MC120 HD color camera connected to a microscope (Europa Promotion Leica DM IL LED, Leica microsystems) with a 10x objective. Scale bar 0.2 mm.

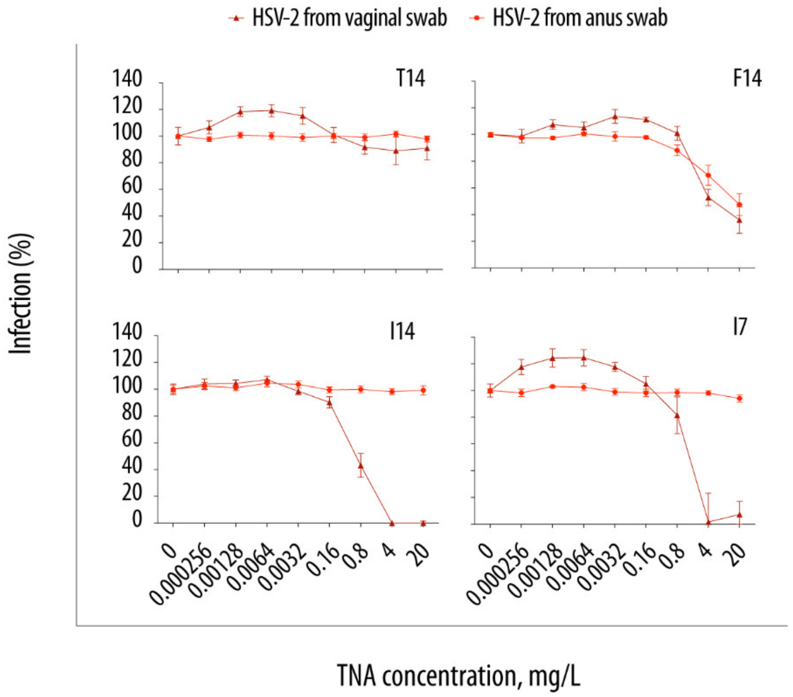

Figure S4. Dose response curves illustrating activity of TNA against clinical HSV-2 isolates sensitive or resistant to acyclovir. Medium of Vero E6 cells were changed to a chemical defined, serum-free medium to avoid extracellular degradation of TNA prior to cell entry. Cells were then incubated with TNAs for 2 h prior. 72 h afterwards infection rates were determined by using MTT. n=2 in triplicates, ±SD

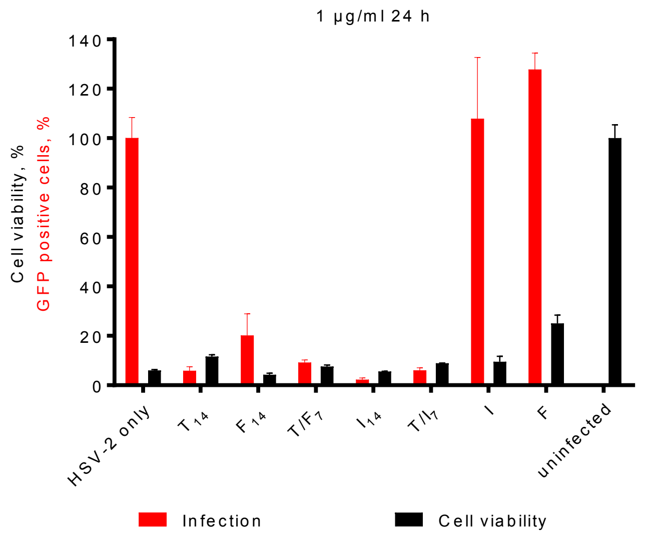

Figure S5. Lipofectamine as a delivery vehicle for TNA has significant toxicity. Vero E6 cells were transfected with lipoplexes (0,5 µl Lipofectamine diluted in 5 µl Opti-Mem medium mixed with 10 µg of TNA diluted in Opti-Mem medium) for thymidine, trifluridine and idoxuridine. Controls were HSV-2 only, idoxuridine (I) and trifluridine (F) without lipoplexes as well as an uninfected sample. Cell viability of treated and infected cells were determined using Celltiter-Glo® assay, GFP positive cells were determined using FACS, both 24 h after infection. n=2 in triplicates, SD.

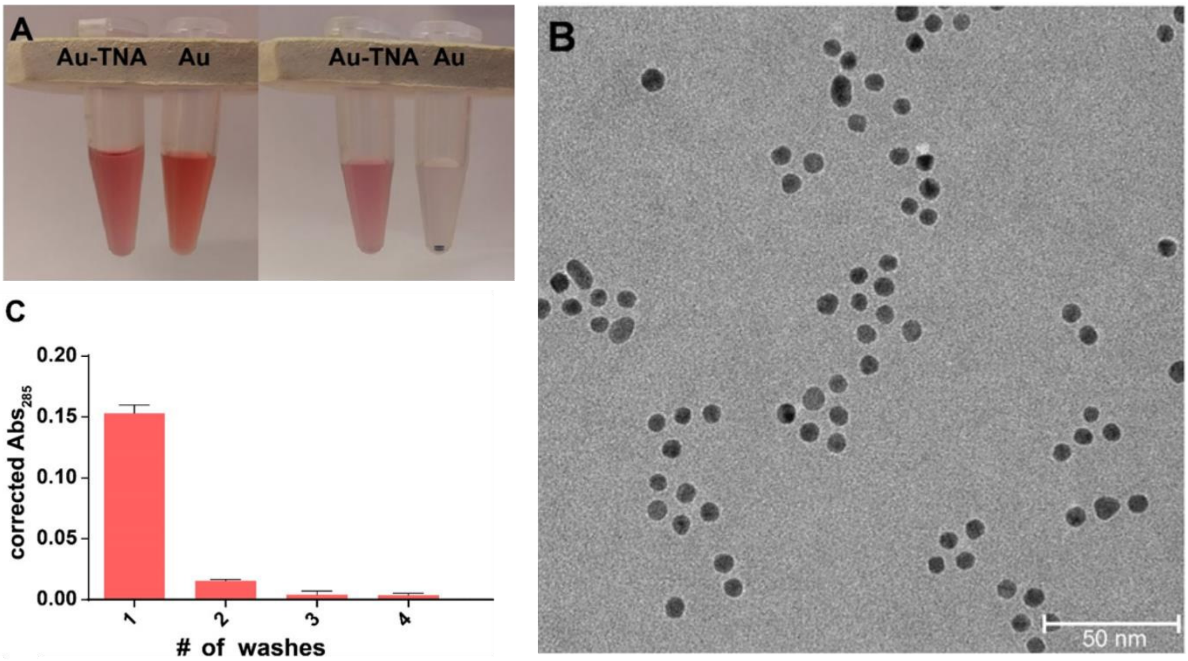

Figure S6. A) TNA-I_{14} coated gold nanoparticles and naked gold nanoparticles before (left) and after (right) 4 centrifugation-resuspension cycles; **B)** Sample TEM image of TNA-I_{14}-coated gold nanoparticles; **C)** UV absorption corresponding to TNAs (I_{14}, 285 nm) of the supernatant liquids in successive washes of the TNA-coated gold nanoparticles revealing the removal of the non-bound TNA.

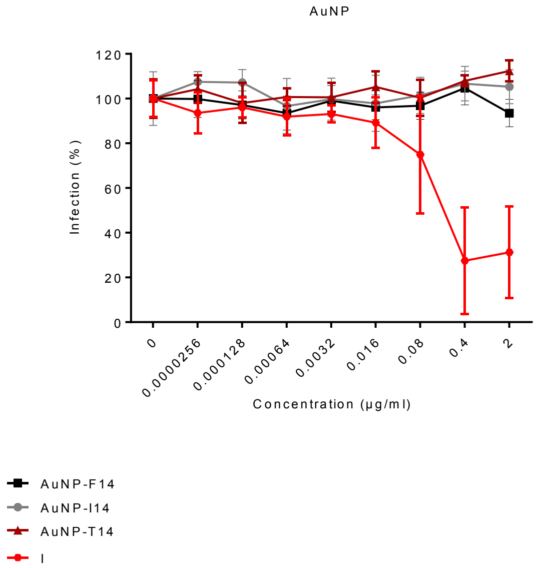

Figure S7. Infectivity of the HSV-2 in Vero E6 cells in the presence of idoxuridine or the Au nanoparticles equipped with TNA. Cytopathic effect and therefore infection was determined using the colorimetric MTT cell viability assay. n=2 in triplicates, SD.

NMR spectra of the synthesized compounds

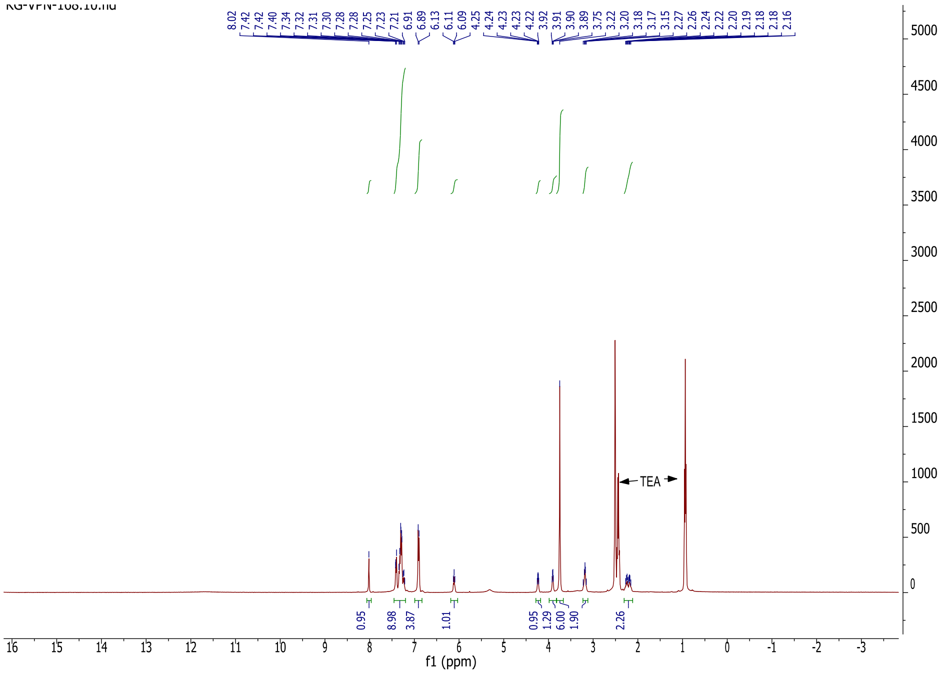

^{1}H NMR spectrum of **3**

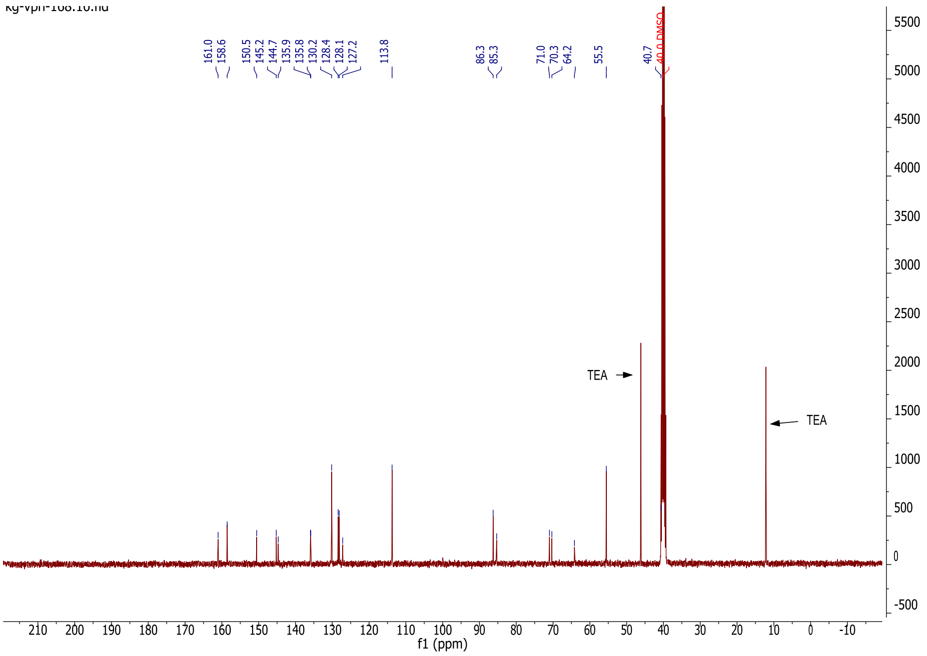

^{13}C NMR spectrum of **3**

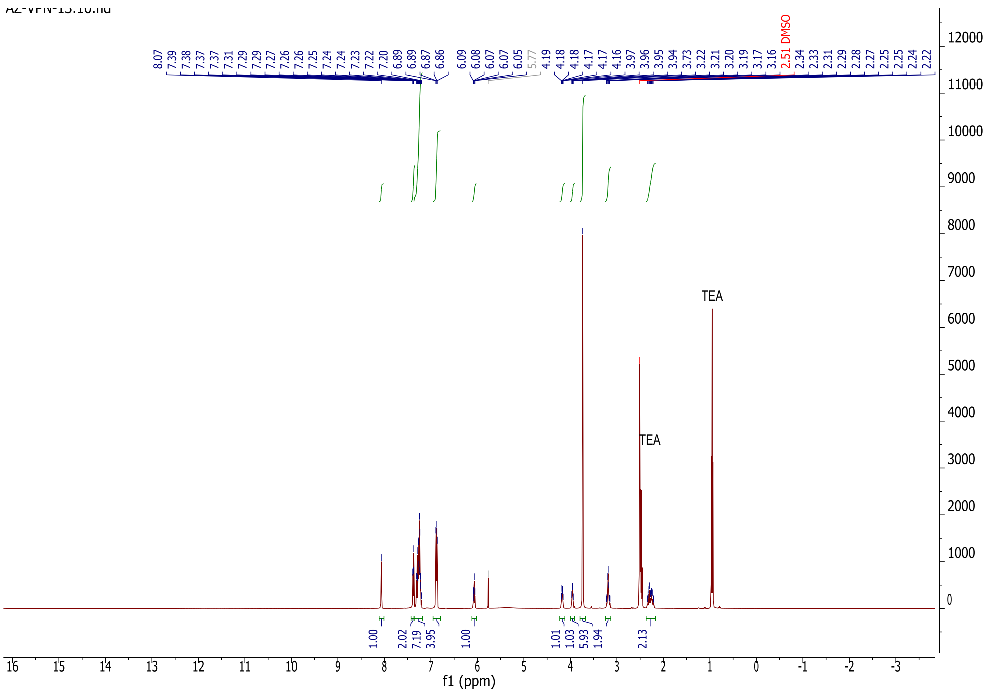

^{1}H NMR spectrum of **4**

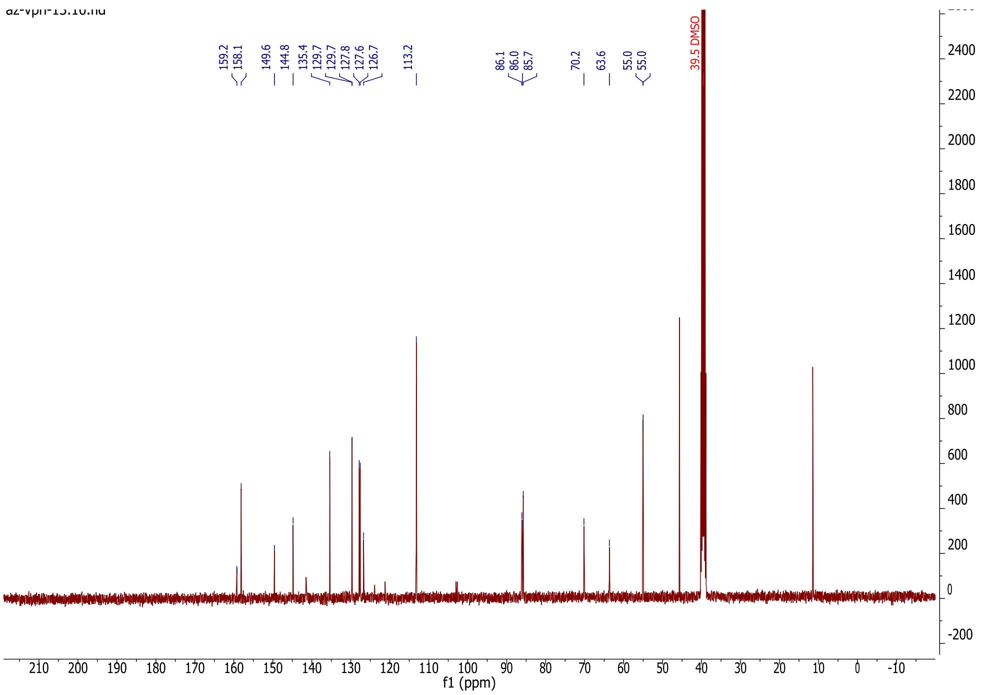

^{13}C NMR spectrum of **4**

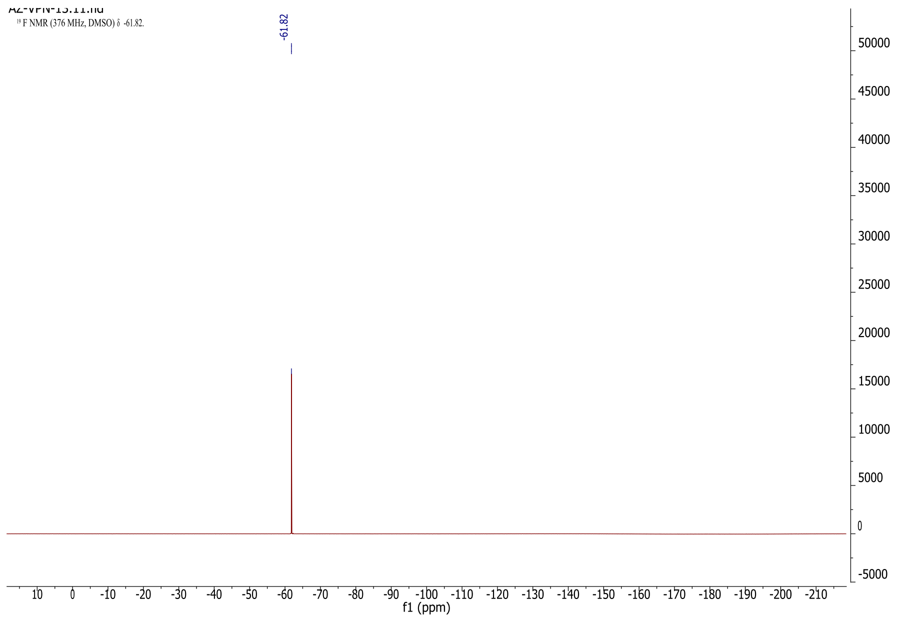

^{19}F NMR spectrum of **4**

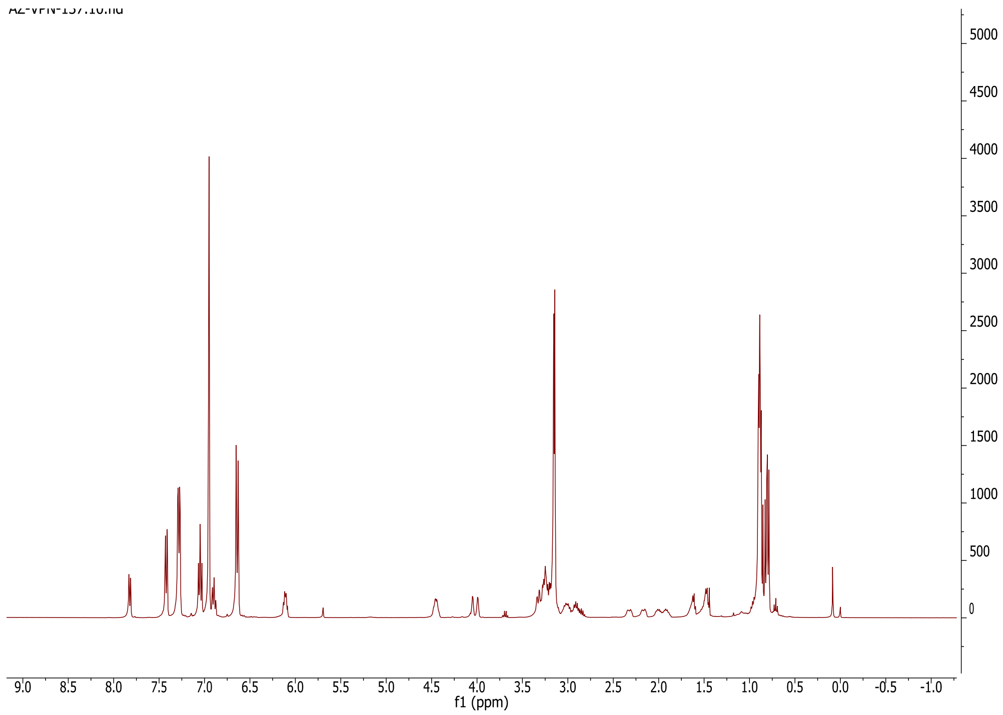

^{1}H NMR spectrum of **5**

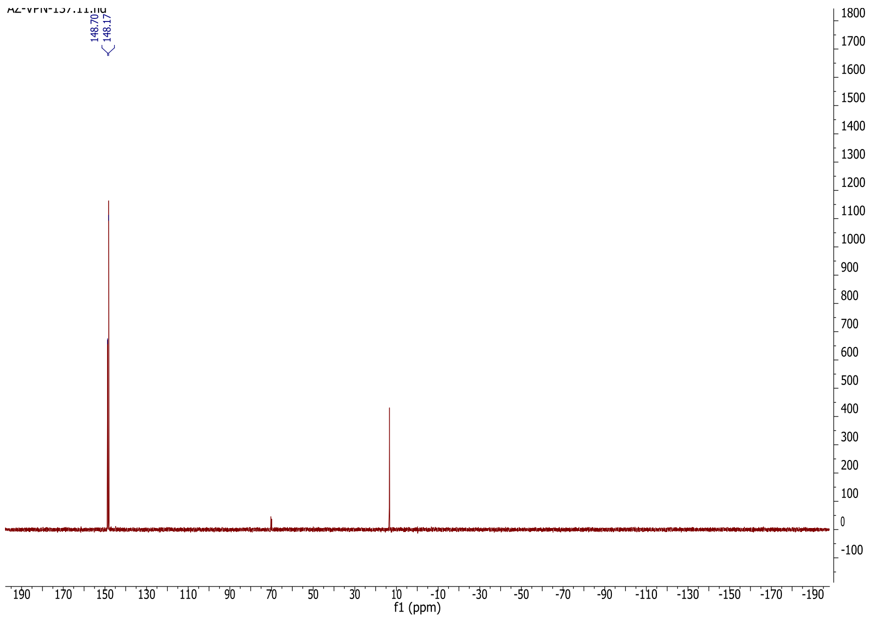

^{31}P NMR spectrum of **5**

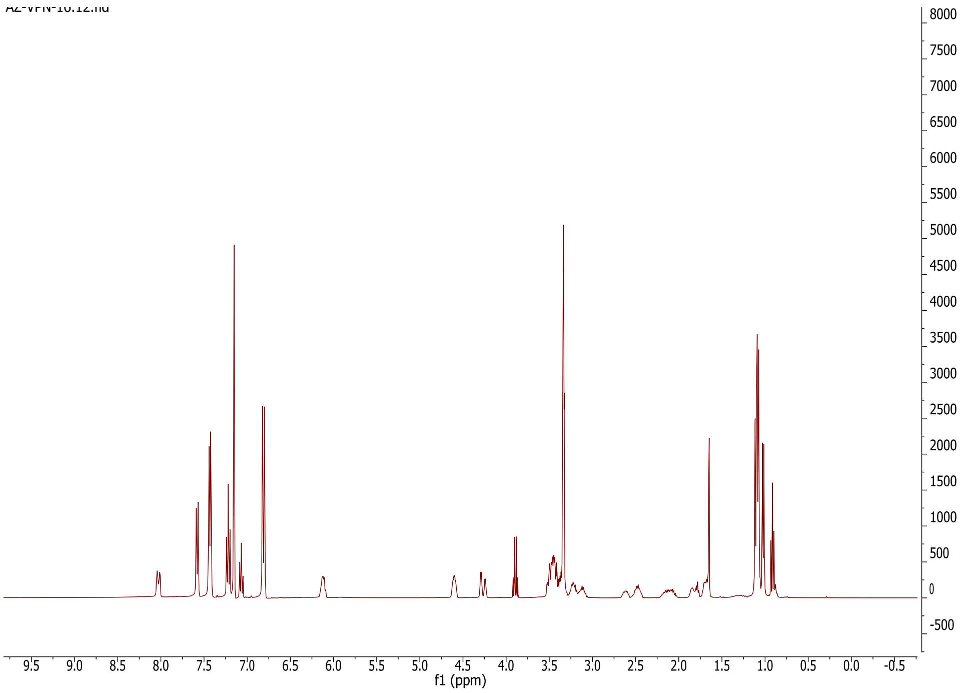

^{1}H NMR spectrum of **6**

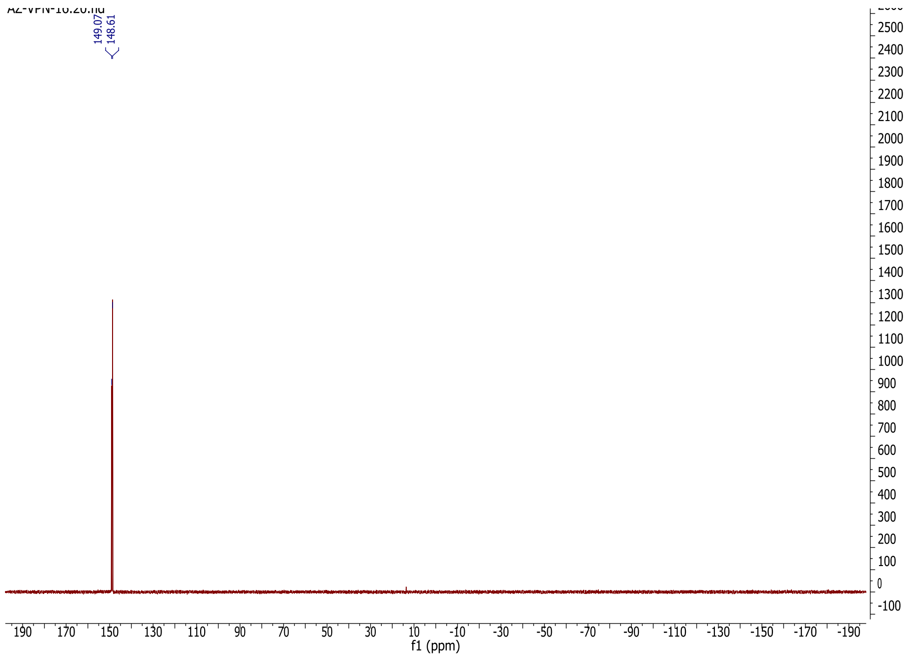

^{31}P NMR spectrum of **6**

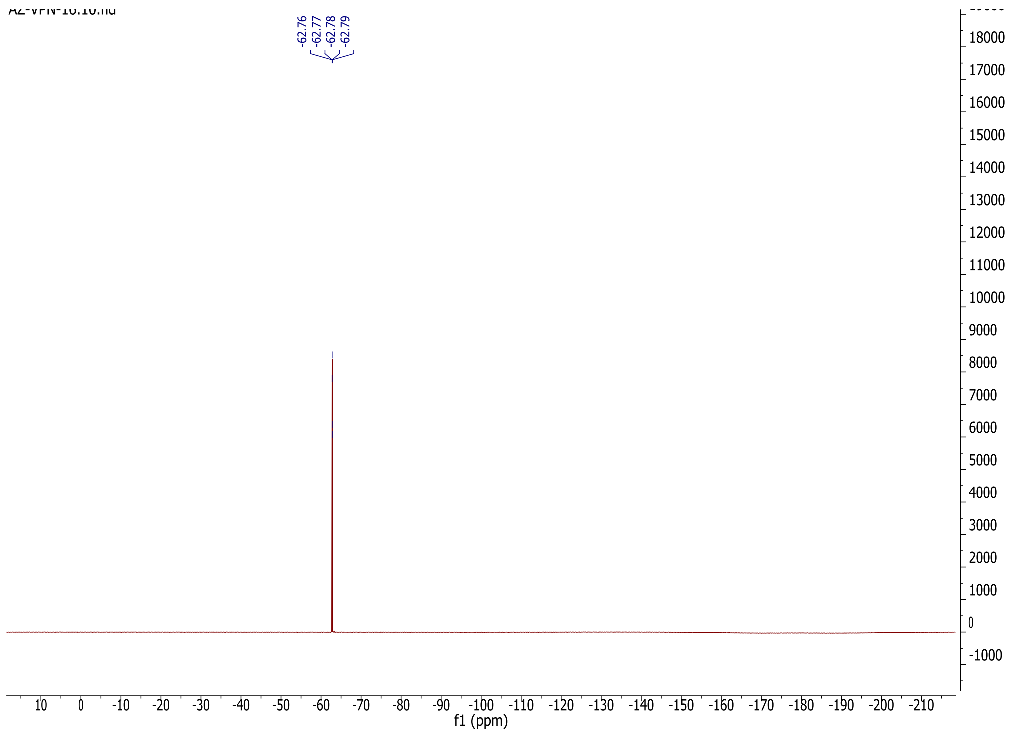

^{19}F NMR spectrum of **6**

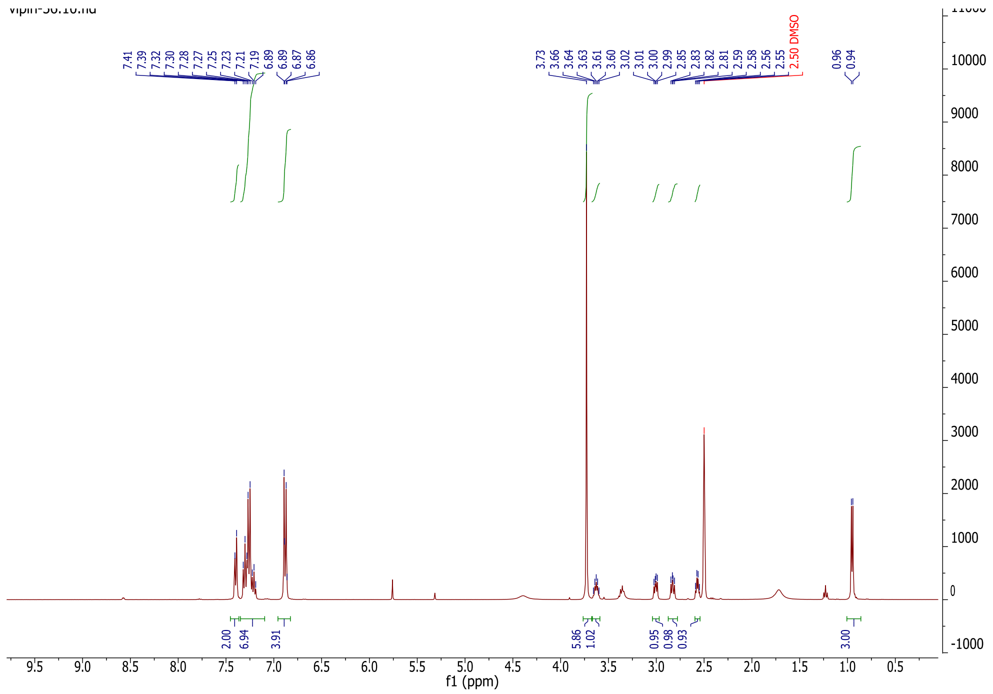

^{1}H NMR spectrum of **9**

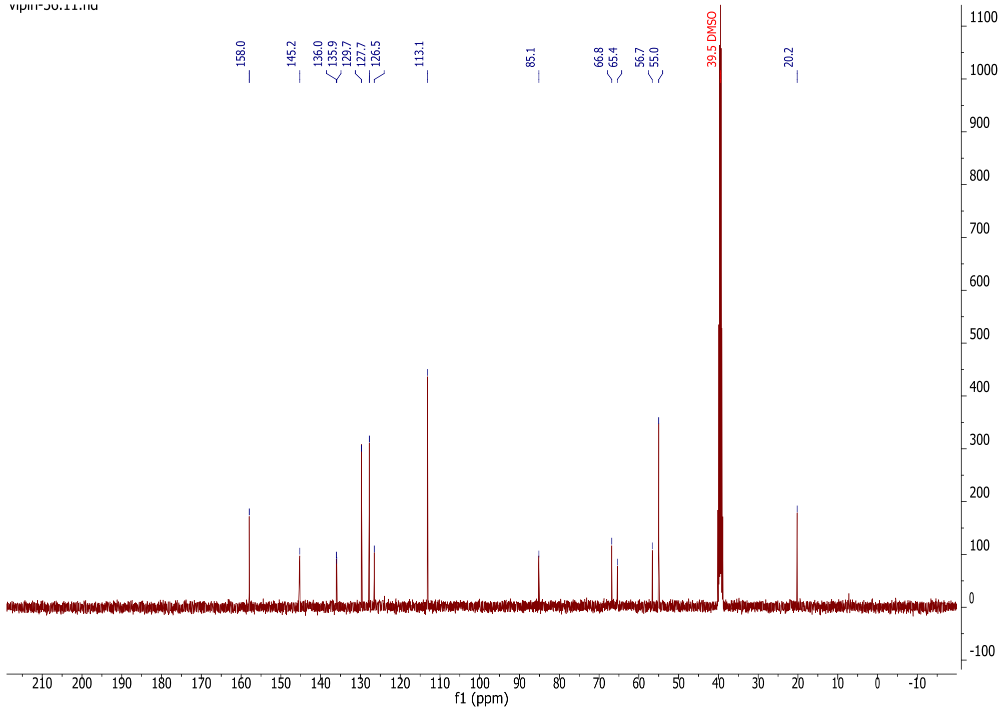

^{13}C NMR spectrum of **9**

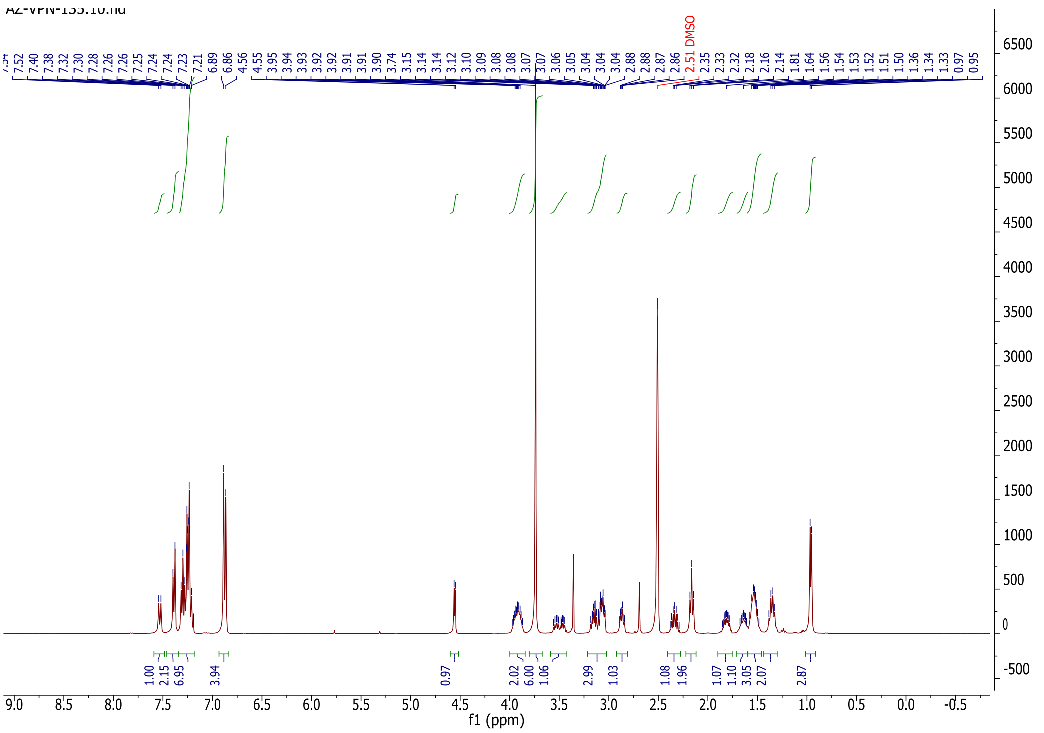

^{1}H NMR spectrum of **11**

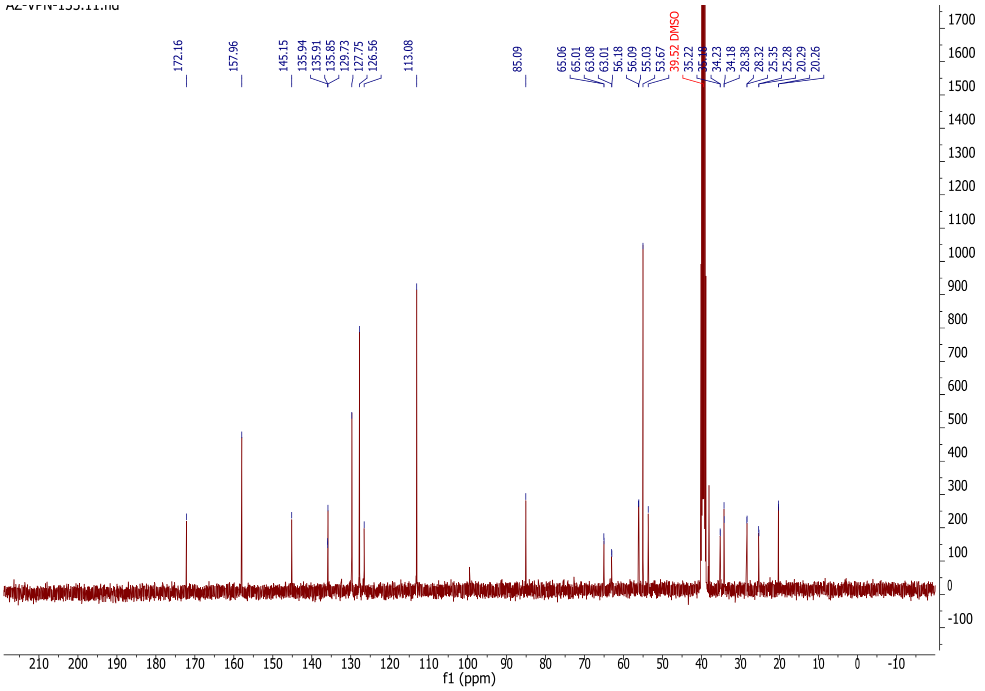

^{13}C NMR spectrum of **11**

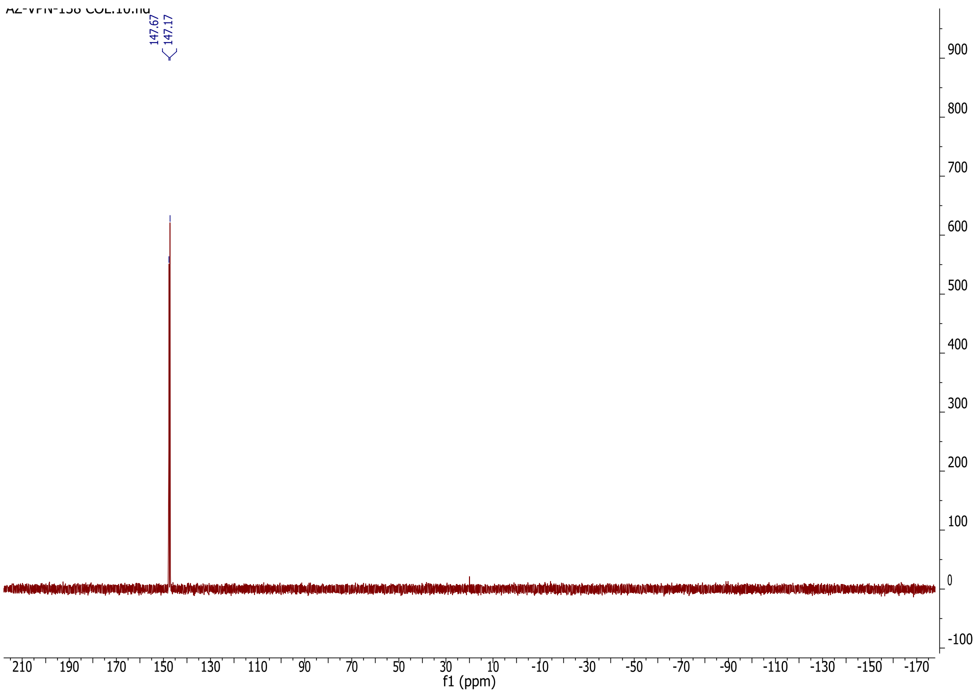

^{31}P NMR spectrum of **12**

Reversed-phase HPLC profiles of oligonucleotides:

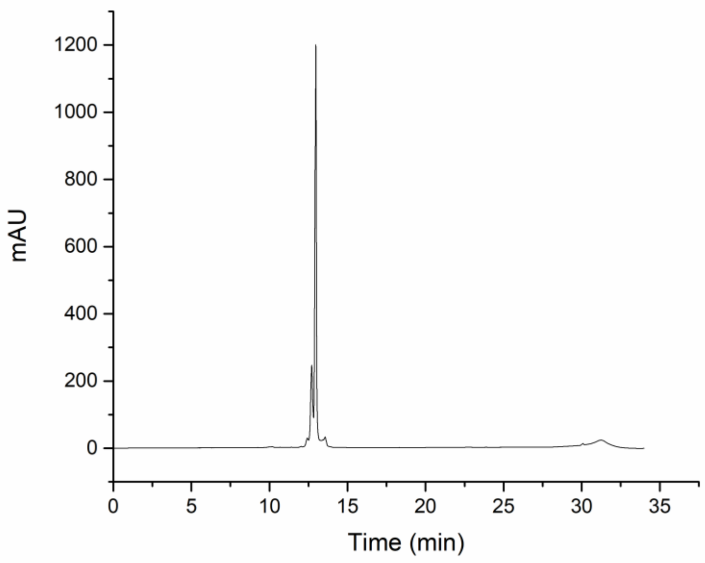

RP-HPLC of VPN-11

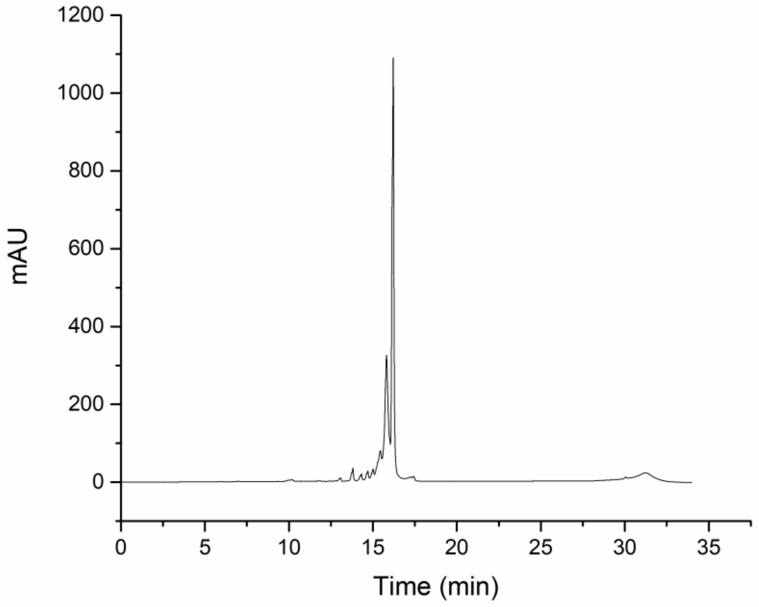

RP-HPLC of VPN-12

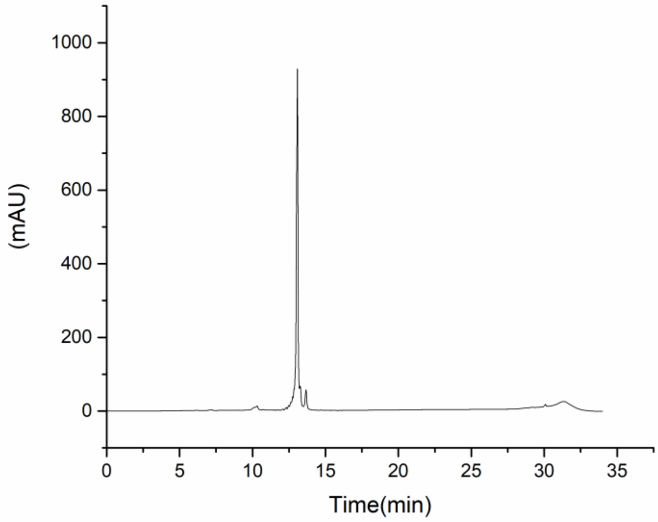

RP-HPLC of VPN-13

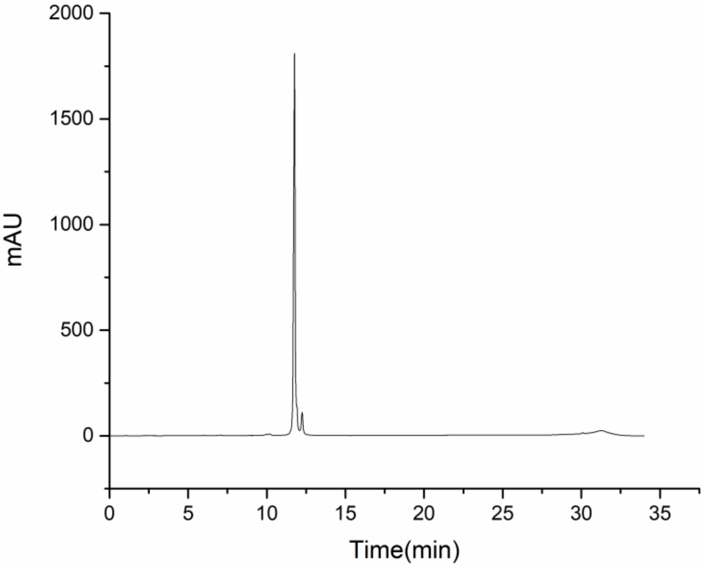

RP-HPLC of VPN-14

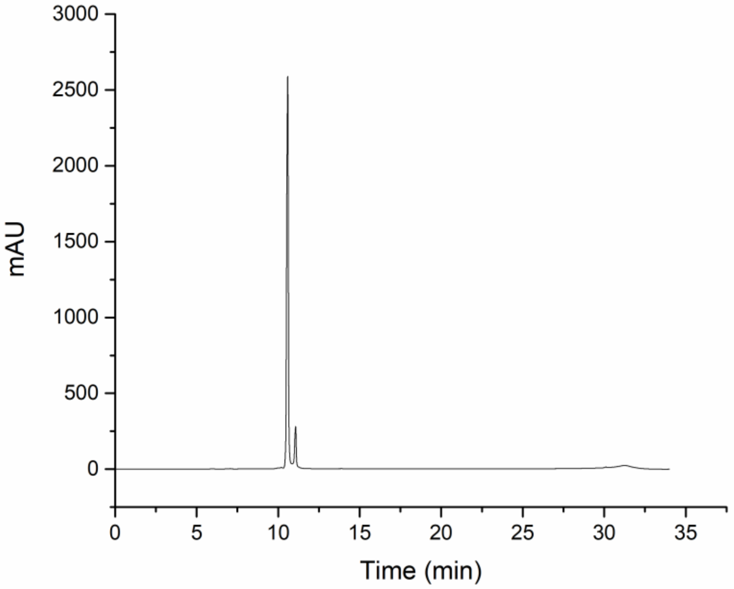

RP-HPLC of VPN-15

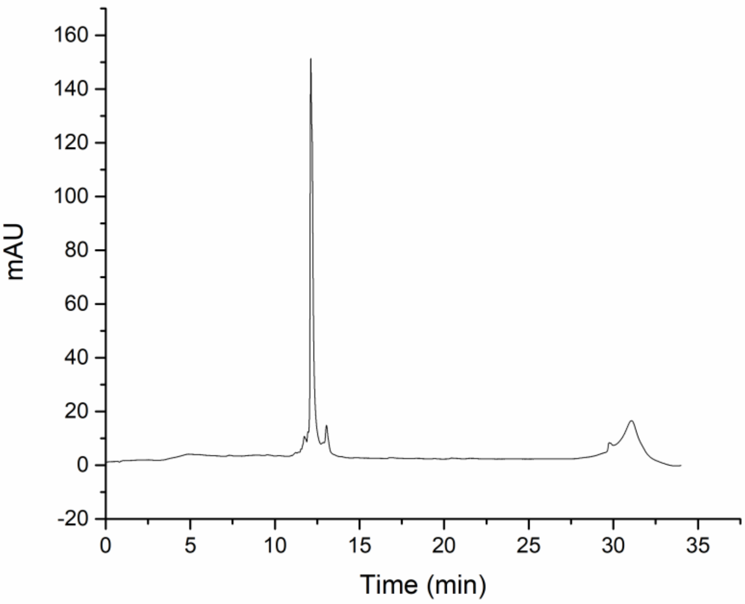

RP-HPLC of VPN-21

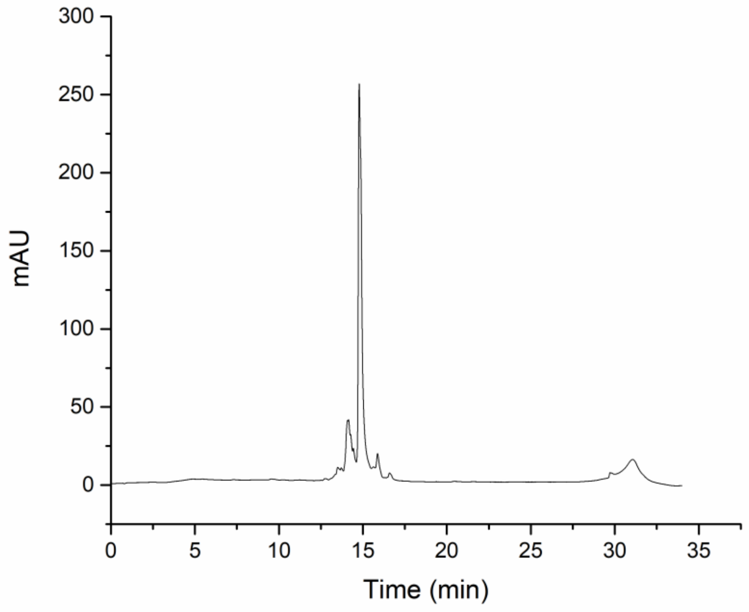

RP-HPLC of VPN-24

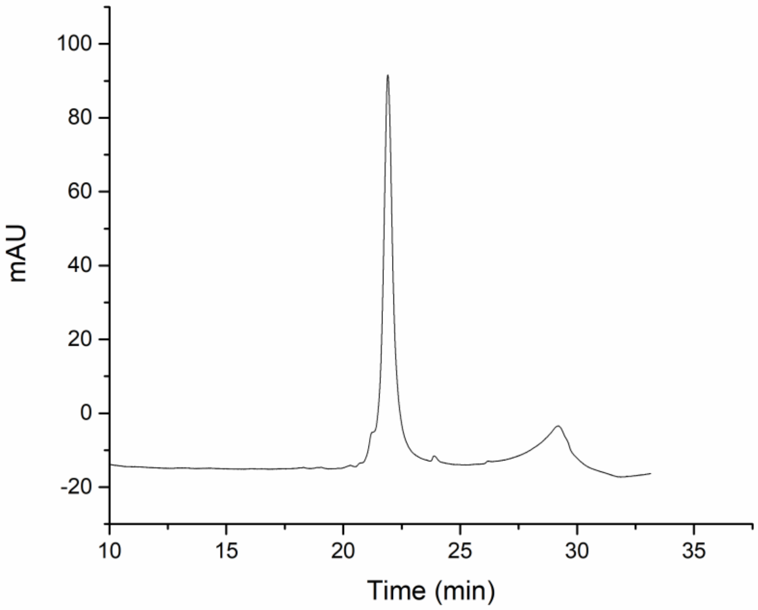

RP-HPLC of VPN-27

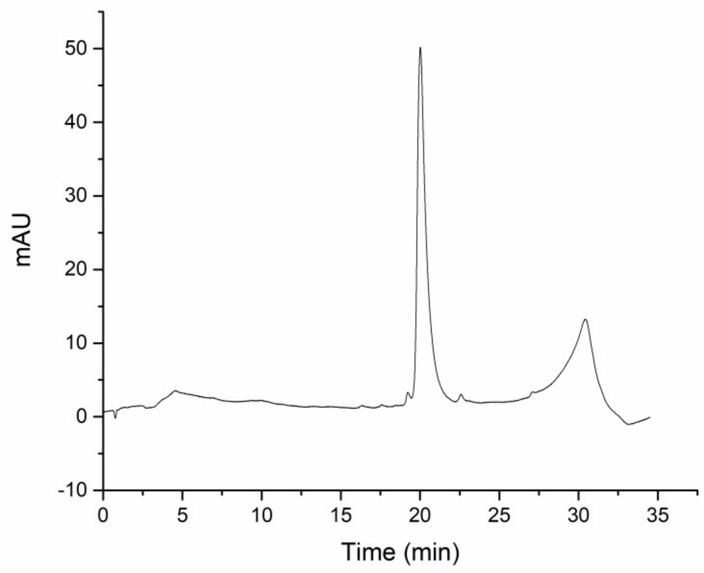

RP-HPLC of VPN-29

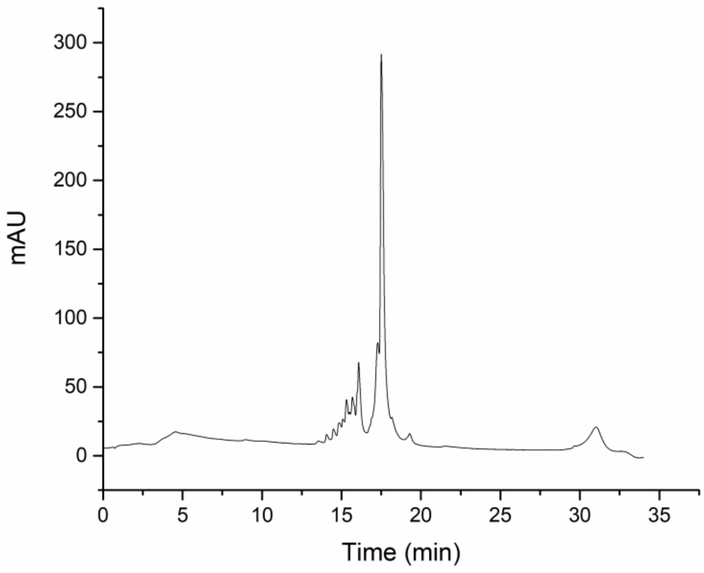

RP-HPLC of VPN-30

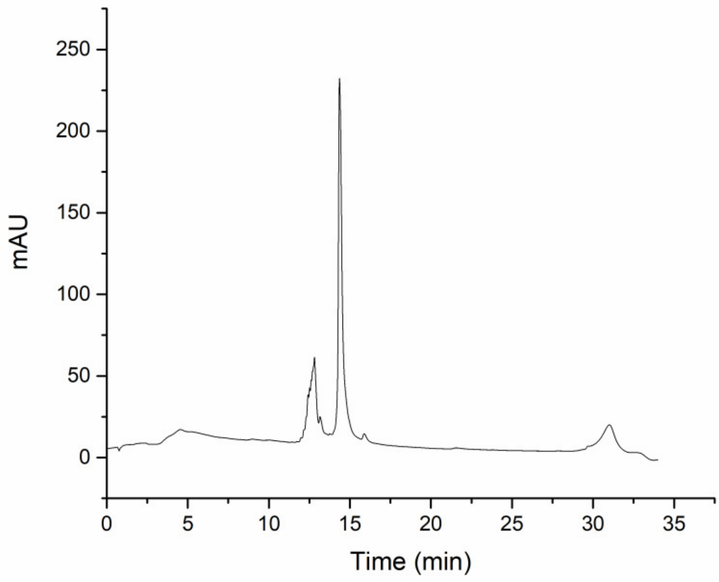

RP-HPLC of VPN-31

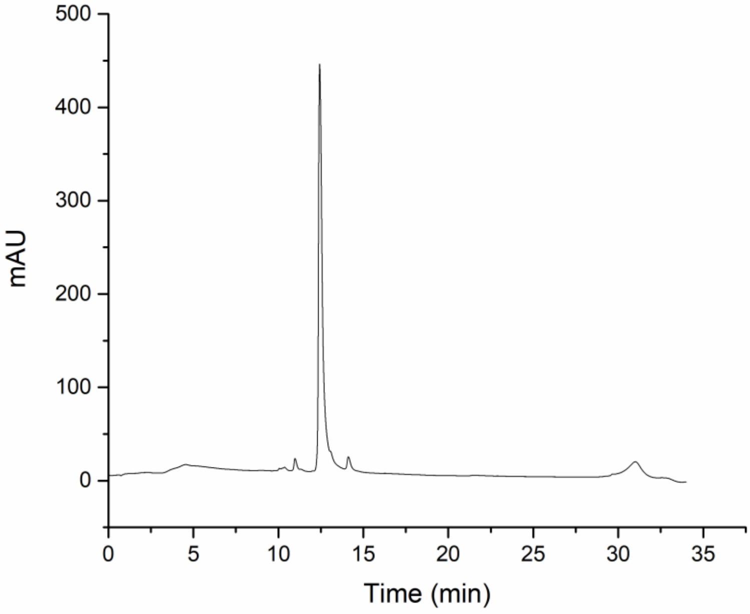

RP-HPLC of VPN-32
